# Use of cast immobilisation versus removable brace in adults with an ankle fracture: multicentre randomised controlled trial

**DOI:** 10.1136/bmj.n1506

**Published:** 2021-07-06

**Authors:** Rebecca Kearney, Rebecca McKeown, Helen Parsons, Aminul Haque, Nick Parsons, Henry Nwankwo, James Mason, Martin Underwood, Anthony C Redmond, Jaclyn Brown, Siobhan Kefford, Matthew Costa, AIR trial collaborators, Jonathan Young, Eamon Ramahandany, Mike Kelly, Nima Heidari, Richard Jeavons, Rajesh Nanda, Carolyn Chadwick, Chris Blundell, Mark Davies, Howard Davies, Raju Aluwhalia, Ines Reichert, Sultan Qasim, Atif Malik, Jordi Ballester, Verity Currall, Simon Burtt, Sandeep Kapoor, Fraser Harrold, Alasdair Macinnes, Harish Karup, Holly Morris, Suranga Giurushihe, Melinda Hav, Abdul Moees, Hemanta Das, Vishal Rajput, Aamir Zubairy, Andrew McAndrew, Rupinderbir Deol, Syed Anjum, Togay Koc, Ahmed Abde Azaz, Zine Beech, Mike Dean, Zoe Lin, Jo Round, Craig White, Yadu Shankarappa, Jit Mangwani

**Affiliations:** 1 Warwick Clinical Trials Unit, University of Warwick, Warwick, UK; 2 Warwick Medical School, University of Warwick, Warwick, UK; 3 Leeds Institute for Rheumatic and Musculoskeletal Medicine, University of Leeds, Leeds, UK; 4 Oxford Trauma and Emergency Care, Nuffield Department of Rheumatology, Musculoskeletal and Orthopaedic Sciences, University of Oxford, Oxford, UK

## Abstract

**Objectives:**

To assess function, quality of life, resource use, and complications in adults treated with plaster cast immobilisation versus a removable brace for ankle fracture.

**Design:**

Multicentre randomised controlled trial.

**Setting:**

20 trauma units in the UK National Health Service.

**Participants:**

669 adults aged 18 years and older with an acute ankle fracture suitable for cast immobilisation: 334 were randomised to a plaster cast and 335 to a removable brace.

**Interventions:**

A below the knee cast was applied and ankle range of movement exercises started on cast removal. The removable brace was fitted, and ankle range of movement exercises were started immediately.

**Main outcome measures:**

Primary outcome was the Olerud Molander ankle score at 16 weeks, analysed by intention to treat. Secondary outcomes were Manchester-Oxford foot questionnaire, disability rating index, quality of life, and complications at 6, 10, and 16 weeks.

**Results:**

The mean age of participants was 46 years (SD 17 years) and 381 (57%) were women. 502 (75%) participants completed the study. No statistically significant difference was found in the Olerud Molander ankle score between the cast and removable brace groups at 16 weeks (favours brace: 1.8, 95% confidence interval −2.0 to 5.6). No clinically significant differences were found in the Olerud Molander ankle scores at other time points, in the secondary unadjusted, imputed, or per protocol analyses.

**Conclusions:**

Traditional plaster casting was not found to be superior to functional bracing in adults with an ankle fracture. No statistically difference was found in the Olerud Molander ankle score between the trial arms at 16 weeks.

**Trial registration:**

ISRCTN registry ISRCTN15537280.

## Introduction

Each year more than 120 000 people in the United Kingdom experience an ankle fracture.^1^ Such fractures represent a major trauma workload, and demand is increasing—because the number of older adults who remain active is increasing, by 2030 ankle fractures are expected to increase threefold.^1^ ^2^ The frequency of this injury places an increasing burden on the UK National Health Service.^3^ Affected adults are unable to engage in usual physical activities for prolonged periods.^1^ ^2^ ^4^ The qualitative impact on individuals is substantial, affecting family and social life, sleep, sense of independence, and psychological wellbeing.^5^

Conventionally, after fracture the ankle is immobilised in a rigid cast for several weeks, which allows the bones to heal but can result in joint stiffness and muscle weakness.^6^ An alternative is a removable brace, which can be taken off to allow early movement. Using a removable brace could prevent the consequences of rigid immobilisation and help to accelerate recovery. Both methods are routinely used in the UK.

A Cochrane review concluded that functional bracing might reduce activity limitation and pain and improve ankle movement. These potential advantages, however, need to be balanced against the increased incidence of adverse events. High quality evidence is not available to support the effectiveness or safety of early movement after ankle fracture.^6^

In light of the increasing numbers of ankle fractures and large personal and societal costs, we compared removable bracing with casting on function, quality of life, and complications in adults with an ankle fracture.

## Methods

This pragmatic, multicentre, superiority randomised controlled trial was undertaken at 20 trauma units in the UK National Health Service. The trial protocol (see supplementary file 1) was accepted for publication on 31 October 2018 and first published online on 18 December 2018.^7^ The independent trial steering committee and data and safety monitoring committee approved the statistical analysis plan. These independent committees were convened to oversee the study throughout the trial’s duration.

### Participants

Trauma research teams at UK NHS trust sites screened adults aged 18 years and older with a closed ankle fracture. In this pragmatic study design, if the treating clinician would usually treat the fracture in a cast for a minimum of three weeks, whether treated non-operatively or operatively, then the patient was considered eligible. Therefore, patients did not meet the initial criteria for entry to the study if the clinician decided that no immobilisation (cast or brace) was required.

On further screening, people were excluded if they had a fracture secondary to known metastatic disease, complex intra-articular fracture (eg, Pilon fracture), wound complications contraindicating a removable bracing, pre-existing neuropathic joint disease, previous ankle fracture already randomised in the present trial, were unable to adhere to trial follow-up procedures, or required close contact casting.^8^

Indications for first line definitive management (operative or non-operative) were according to usual practice for each individual clinician, as were any adjunctive treatments (eg, antibiotic use). The trial population included patients managed both operatively and non-operatively.

Operatively managed participants had initially received standard local clinical care in the UK, typically consisting of a temporary partial backslab, and wound check about two weeks after surgery. At this point in the care pathway, randomisation processes were completed and a member of the clinical team applied the intervention.

Non-operatively managed participants completed the randomisation processes immediately at presentation to the trauma service, when a member of the clinical team then applied the cast or removable brace. As it is usual clinical practice in the UK to delay decisions about first line non-operative management (ie, to check if the fracture is stable) for some patients, our eligibility criteria allowed up to three weeks before a definitive decision was required.

### Randomisation and blinding

Baseline data were collected before randomisation. Participants were randomised on a 1:1 basis to the two study intervention arms sequentially as they presented. A secure web based system maintained by an independent randomisation team allocated participants using a minimisation algorithm with a random element and stratification by centre, operative or non-operative management, and age (≤49 *v* ≥50 years).^1^ ^9^

Blinding of participants and clinicians was not possible. All follow-up data were participant reported through postal questionnaires; no clinicians or researchers assessed outcome measures.

### Interventions

The interventions were worn for a minimum of three weeks in both groups.

Standard below knee cast immobilisation was applied according to local procedures. Participants in the cast group started active unloaded ankle range of movement exercises once the cast was removed.

Removable braces were of a fixed angle design, to replicate what is routinely used in UK practice, and were applied in accordance with local procedures. The specific brand of removable brace was not standardised across sites, and each site used its own brand. The treating clinician in the trauma service setting encouraged participants in verbal and written formats to take off the removable brace to complete active unloaded ankle range of movement exercises as often as pain allowed, with a recommendation for each movement to be performed 10 times, three times a day (see supplementary file 2).

In this pragmatic trial, any other rehabilitation input beyond rigid immobilisation in a cast compared with early active movement in a removable brace was at the discretion of the treating member of the clinical team, in accordance with current UK practice. This included choice of weightbearing for the participant, duration of immobilisation period (beyond the minimum of three weeks), and decision to onward refer participants to physiotherapy services. These details were collected for each participant as part of the trial dataset.

### Outcome measures

The primary outcome was the Olerud Molander ankle score at 16 weeks; a self-administered questionnaire that consists of nine different items (pain, stiffness, swelling, stair climbing, running, jumping, squatting, supports, and work or activities of daily living).^10^ Scores range from 0 for totally impaired to 100 for completely unimpaired.

We chose the primary outcome time point of 16 weeks after consultation with academics, clinicians, and public and patient representatives. This time point was based on previous trials of ankle fracture, which found that the steepest recovery occurs in the first four months of injury.^8^ Additional longer term secondary data are being collected at 6, 12, 18, and 24 months as part of a preplanned longer term follow up, to be reported separately to capture any later stage complications.

Secondary outcomes were complications (deep vein thrombosis, pulmonary embolism, pain, swelling, numbness around the foot, wound infection, and fracture healing), resource use, and self-administered health related quality of life measures (EQ-5D-5L) and leg specific functional scores (Manchester-Oxford foot questionnaire and disability rating index).^11^ ^12^ ^13^ All data were collected at 6, 10, and 16 weeks, except for the Manchester-Oxford foot questionnaire, which was collected at 16 weeks only. All follow-up data were collected through postal questionnaires returned to the central trial team.

### Statistical analysis

The target between group difference for the primary Olerud Molander ankle score outcome was 10 points, consistent with other studies of ankle fracture management—this is the accepted minimally clinically important difference.^8^ ^14^ The standard deviation of the Olerud Molander ankle score from previous feasibility work was 28 points, so we used a conservative estimate of 30 points for the sample size calculation.^15^ With significance set at the 5% level and at 90% power, we determined that we needed data on 382 participants. Allowing for 20% loss to follow up, 478 participants were required. As recruitment exceeded planned expectations, to improve precision of our effect size estimate we obtained relevant approvals to continue recruiting to the predefined end recruitment date. The study was not powered to detect differences in the secondary outcome measures.

The primary analysis was the Olerud Molander ankle score at 16 weeks. The planned main analysis, which was on an intention-to-treat basis, used adjusted mixed effects linear regression analysis to assess evidence for differences in the Olerud Molander ankle score between the intervention arms. We included study recruitment centre as a random effect and adjusted the analysis for the stratification variables (age and operative or non-operative management) as fixed effects. Participants who maintained their allocated intervention for at least three weeks were considered to have followed the protocol. A sensitivity analysis to investigate the effect of missingness and adherence was conducted using multiple imputation.^16^ All analyses were implemented in R.^17^

Two prespecified subgroup analyses were undertaken to determine whether the intervention effect differed between participants who received operative or non-operative treatment before the study intervention and between those who were younger than 50 years or 50 years and older at randomisation; those older than 50 years being more likely to have experienced a fragility fracture related to osteoporosis. The original cut-off for 60 years was amended to 50 years following advice from the independent oversight committees but before publication of the final protocol^7^ and approval of the statistical analysis plan. The subgroup analyses followed the methods described for the primary analysis, with additional interaction terms incorporated into the mixed effects model.

### Patient and public involvement

Public and patient representatives co-produced this study. After a single site feasibility trial funded by the National Institute for Health Research (ISRCTN17809322),^15^ two public and patient representatives (RG and KK) continued their roles through the planning, development, and delivery of the current main randomised controlled trial.

## Results

Between 9 October 2017 and 30 September 2019, 20 NHS trusts screened 3144 adults with an ankle fracture, of whom 1152 were not eligible. Of the remaining 1992 (35%) participants, 669 were randomised: 334 to a cast and 335 to a removable brace. Six hundred and twenty seven of the remaining 1323 patients (31%) did not want to take part in the trial (428 had preference for a specific treatment, 79 did not want to be part of a research study, and 90 had other reasons) and for the other 696 (35%) patients the treating clinician did not offer the option to take part (579 had preference for a specific treatment and 117 had other reasons).

**Fig 1:**
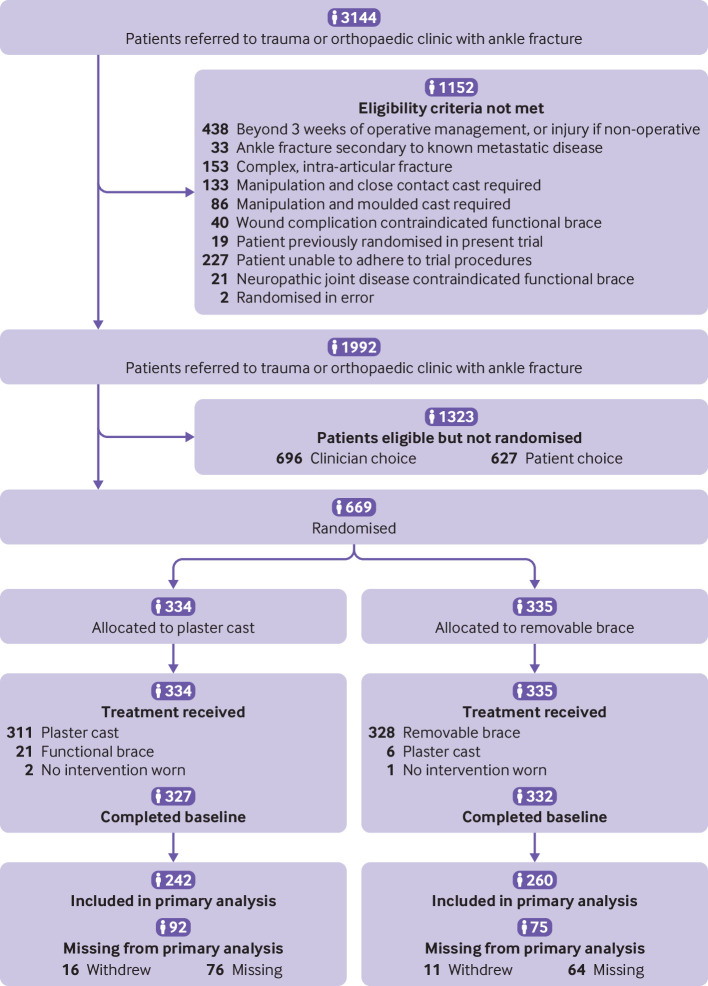
Trial profile

Twenty seven participants withdrew before the primary outcome point of 16 weeks. Five hundred and two participants completed the primary outcome Olerud Molander ankle score score (75%) and were included in the final analysis (fig 1).

The mean age of participants was 46.3 years (SD 17 years) and more than half the participants were women (57%). All participants had a clear ankle fracture on a radiograph, with 624 (93%) showing lateral malleolar involvement, 194 (29%) medial malleolar involvement, and 120 (18%) posterior malleolar involvement. Ankle fracture in 428 (64%) participants was due to a low energy fall. Surgery was performed in 54% of the randomised participants. The groups were well balanced across all baseline characteristics (table 1).

**Table 1:** Baseline characteristics of adults with ankle fracture allocated to plaster cast or removable brace in intention-to-treat population*

Characteristics	Cast (n=334)	Removable brace (n=335)	Overall (n=669)
Sex:			
Women	198 (59)	183 (55)	381 (57)
Men	136 (41)	152 (45)	288 (43)
Ethnicity:			
Asian	15 (5)	12 (4)	27 (4)
Black, African, and Caribbean	15 (5)	14 (4)	29 (4)
Mixed race	7 (2)	8 (2)	15 (2)
Other	47(2)	6 (2)	13 (2)
White	289 (87)	289 (88)	581 (87)
Mean (SD) pre-injury OMAS score	93.4 (16)	93.4 (16)	93.4 (16)
Mean (SD) baseline (post-injury) OMAS score	20.8 (17)	21.2 (18)	21.0 (17)
Mean (SD) age (years)	46.7 (17)	45.9 (16)	46.3 (17)
Age category (years):			
≤49	185 (55)	186 (56)	371 (55)
≥50	149 (45)	149 (44)	298 (45)
Mean (SD) body mass index	28.2 (6)	28.6 (6)	28.4 (6)
Mechanism of injury†:			
Low energy fall	216 (64)	212 (63)	428 (64)
High energy fall	49 (15)	60 (18)	109 (16)
Road traffic incident	14 (4)	12 (4)	26 (4)
Crush injury	1 (<1)	1 (<1)	2 (<1)
Sports injury	26 (8)	23 (7)	49 (7)
Other	32 (10)	29 (8)	61 (9)
Side of injury:			
Right	158 (47)	170 (51)	328 (49)
Left	176 (52)	161 (48)	337 (50)
Malleolus involvement†:			
Lateral	310 (93)	314 (94)	624 (93)
Medial	110 (33)	84 (25)	194 (29)
Posterior	60 (18)	60 (18)	120 (18)
Fracture management:			
Operative	182 (55)	182 (54)	364 (54)
Non-operative	152 (45)	153 (46)	305 (46)
Advised weightbearing:			
Full	110 (33)	109 (33)	219 (33)
Partial	65 (20)	75 (22)	140 (21)
None	157 (47)	146 (44)	303 (45)
Concurrent injuries:			
No	307 (92)	317 (95)	624 (93)
Yes	27 (8)	18 (5)	45 (7)
Regular smoker:			
No	268 (80)	259 (77)	527 (79)
Yes	64 (19)	71 (21)	135 (20)
Alcohol/week (units):			
0-7	233 (70)	205 (61)	438 (65)
8-14	50 (15)	71 (21)	121 (18)
15-21	31 (9)	33 (10)	64 (10)
>21	20 (6)	24 (7)	44 (7)
Concurrent drugs:			
Steroids	14 (4)	10 (3)	24 (4)
Other	224 (67)	215 (64)	439 (66)
Diagnosis before injury:			
Diabetes	16 (5)	16 (5)	32 (5)
Leg fracture (past 12 months)	3 (1)	2 (1)	5 (1)
Injury to leg (past 12 months)	2 (1)	4 (1)	6 (1)

OMAS=Olerud Molander ankle score.

* <3% missing in any category.

† Multiple categories possible.

Twenty three participants in the cast group (n=334) did not adhere to the treatment allocation and crossed over to the removable brace group (20 owing to participant preference). Seven participants in the removable brace group (n=335) did not adhere to the treatment allocation and crossed over to the cast group (four owing to participant preference; see supplementary file 3, table 4).

Other rehabilitation input beyond rigid immobilisation in a cast versus early active movement in a removable brace was at the discretion of the treating member of the clinical team. This input was balanced across the groups. The number of onward referrals to physiotherapy was similar between the groups: 182 in the cast group and 166 in the removable brace group (see supplementary file 3).

No statistically significant difference was found in the Olerud Molander ankle score at 16 weeks, using the primary adjusted intention-to-treat analysis (mean difference 1.8, 95% confidence interval −2.0 to 5.6, favours brace). Nor was a clinically relevant difference found at the six week and 10 week time points (table 2). No clinically relevant differences were found in the disability rating index, Manchester-Oxford foot questionnaire, or EQ-5D-5L secondary outcomes at any time point (table 3). 

**Table 2:** Olerud Molander ankle score (OMAS) in adults with ankle fracture allocated to plaster cast or removable brace in intention-to-treat population*

	Cast (n=334)			Removable brace (n=335)			Between group difference (95% CI)		
	No	Mean (SD) OMAS		No	Mean (SD) OMAS		Unadjusted	Adjusted†	P value
6 weeks	241	37.2 (22.1		256	39.6 (20.6)		2.4 (−1.4 to 6.2)	2.2 (−1.4 to 5.8)	0.23
10 weeks	229	47.1 (21.7)		239	51.5 (23.0)		4.5 (0.4 to 8.5)	4.5 (0.6 to 8.3)	0.02
16 weeks	242	62.4 (23.4)		260	64.5 (22.4)		2.1 (−1.9 to 6.2)	1.8 (−2.0 to 5.6)	0.35

* Positive values in favour of removable brace.

† Estimates are from linear regression model adjusted for patient sex, age group, and fracture management at baseline.

**Table 3:** Secondary outcomes in adults with ankle fracture allocated to plaster cast or removable brace in intention-to-treat population*

	Cast (n=334)			Removable brace (n=335)			Between group difference (95% CI)		
	No	Mean (SD)		No	Mean (SD)		Unadjusted	Adjusted†	P value
DRI:									
6 weeks	222	57.7 (20.6)		247	51.7 (22.6)		−6.0 (−9.9 to −2.1)	−5.6 (−9.4 to −1.8)	0.004
10 weeks	218	43.8 (22.5)		229	38.8 (23.4)		−5.0 (−9.3 to −0.8)	−5∙0 (−9.2 to −0.9)	0.01
16 weeks	213	32.8 (23.9)		235	31.4 (24.7)		−1∙5 (−6.0 to 3.0)	−1.0 (−5.4 to 3.4)	0.65
EQ-5D-5L:									
6 weeks	241	0.497 (0.272)		258	0.534 (0.258)		0.037 (−0.010 to 0.084)	0.036 (−0.010 to 0.082)	0.12
10 weeks	228	0.647 (0.192)		239	0.66 (0.18)		0∙013 (−0.021 to 0.047)	0.013 (−0.020 to 0.047)	0.43
16 weeks	241	0.702 (0.198)		259	0.73 (0.177)		0∙028 (−0.005 to 0.061)	0.026 (−0.006 to 0.058)	0.11
MOXFQ:									
16 weeks	218	38.9 (24.7)		233	36.9 (23.6)		−2.0 (−6.5 to 2.5)	−1.32 (−5.6 to 3.0)	0.54

DRI=disability rating index; EQ-5D-5L=self-administered health related quality of life measures; MOXFQ=Manchester-Oxford foot questionnaire.

* Positive values in favour of removable brace.

† Fixed effect model accounting for sex, age group, and fracture management only. Random effect model did not improve fit, hence omitted.

The results of secondary unadjusted and per protocol analyses were not materially different from those of the primary analysis. The study was not, however, powered to detect a difference in secondary outcome data. No evidence was found in predefined subgroup analyses that effects differed according to age or whether or not the participant had surgical treatment (see supplementary file 3, table 6).

As loss to follow-up was higher than the expected 20% at the primary analysis point, multiple imputation using chain equations was used to investigate the robustness of the results. Missingness of the primary outcome was not evenly distributed between the groups, with more data missing in the cast group (completeness: cast group 73%; removable brace 78%).

Twenty five imputed datasets were created for each model and the coefficients were pooled using Rubin’s rules used to combine estimates. Missing data on the Olerud Molander ankle score were imputed using a predictive mean matching method. Variables were chosen from the baseline and randomisation set on the basis of association with missingness at 16 week follow-up. Predictor variables chosen were fractures on medial and posterior malleolus, concurrent injuries on the same leg, body weight, smoking status, age group, sex, and fracture management.

Sensitivity analyses on the imputed dataset gave similar results to those of the primary analysis, suggesting that missingness did not impact on the interpretation of the primary analysis (see supplementary file 3, table 3).

Complications were similar across the two groups. Important complications in the cast group were deep vein thrombosis (n=3), pulmonary embolism (n=1), chronic regional pain syndromes (n=2), and further surgery (n=4)—revision surgery for failed primary fixation (n=1), elective removal of metal work (n=2), and removal of metal work secondary to infection (n=1). Important complications in the removable brace group were deep vein thrombosis (n=3), pulmonary embolism (n=1), chronic regional pain syndrome (n=1), problems with fracture healing (n=1), and further surgery (n=8)—revision surgery for failed primary fixation (n=1), elective removal of metal work (n=4), and removal of metal work secondary to infection (n=3; table 4).

**Table 4:** Analysis of secondary outcome complications from baseline to 16 weeks in adults with ankle fracture allocated to plaster cast or removable brace in intention-to-treat population.* Values are numbers (percentages) unless stated otherwise

	Cast (n=334)	Removable brace (n=335)	Odds ratio (95% CI)	P value†
Wound infection requiring antibiotics‡	10 (5.5)	19 (10.4)	2.0 (0.9 to 5.0)	0.12
Wound breakdown or dehiscence‡	7 (3.8)	15 (8.2)	2.2 (0.8 to 6.7)	0.12
Further surgery for ankle fracture‡	4 (2.2)	8 (4.4)	1.8 (0.5 to 7.1)	0.41
Pressure sore or ulcer	10 (3.0)	6 (1.8)	0.6 (0.17 to 1.8)	0.32
Numbness at side of foot	51 (15.3)	42 (12.5)	0.8 (0.5 to 1.3)	0.31
Non-union of fracture	0	1 (0.3)	NA	1.00
Deep vein thrombosis	3 (0.9)	3 (0.9)	1 (0.1 to 7.5)	1.00
Pulmonary embolism	1 (0.3)	1 (0.3)	1 (0.0 to 78.4)	1.00
Chronic regional pain syndrome	2 (0.6)	2 (0.6)	1 (0.1 to 13.8)	1.00

NA=not applicable.

* Numbers represent complications reported at least once by each participant.

† Fisher’s exact test.

‡ Numbers are only applicable to those who had operative management: cast (n=182) removable brace (n=182).

## Discussion

This randomised controlled trial found no statically significant difference in ankle function between a plaster cast and a removable fixed angle brace at 16 weeks in adults with an ankle fracture. The upper limit of the 95% confidence interval effectively excluded any possibility that one intervention was superior to the other.

No clinically relevant differences were found in Olerud Molander ankle score at secondary time points or in the secondary outcome measures of Manchester-Oxford foot questionnaire, disability rating index, quality of life, and complications. We found no statistically significant differences in the safety profiles of serious complications across both interventions. A higher number of complications occurred in the removable brace group, particularly wound breakdown (7 *v* 15), wound infection (10 *v* 19), and need for further surgery (4 *v* 8).The study was not, however, powered to detect a difference in these secondary outcomes.

### Comparison with other studies

A Cochrane review identified 10 randomised controlled trials that compared a removable type of immobilisation and early movement with a cast and no early movement (n=531).^6^ In our updated search, we identified four subsequent randomised controlled trials (n=451).^14^ ^15^ ^18^ ^19^ One trial (n=50) was the Ankle Injury Rehabilitation feasibility study to this current study and made no inferences to clinical effectiveness.^15^ A further study (n=110) concluded that removable bracing was superior at six weeks, but the difference on the Olerud Molander ankle score diminished by 12 weeks.^18^ These findings were supported by a further randomised controlled trial that used a visual analogue scale pain score as a primary measure (n=44).^14^ The largest randomised controlled trial (n=247) indicated non-inferiority of removable bracing at 6, 12, and 52 weeks using the Olerud Molander ankle score.^19^ No trials found a difference in safety profiles of removable bracing compared with cast.

This trial did not find any clinically relevant differences between traditional cast compared with removable brace management at any time point, in keeping with previous trials.

### Strengths and limitations of this study

The current trial, a large scale pragmatic randomised controlled trial, was designed to evaluate the clinical effectiveness of a plaster cast versus removable bracing for the management of ankle fractures in adults. The main limitation of our study was the 25% loss to follow-up; however, the minimum sample size was exceeded by a large margin and our post hoc sensitivity analysis, accounting for missing data, produced similar results to the primary analysis, providing reassurance that the primary analysis was robust. Bias could potentially have been introduced because participants who were lost to follow-up might have been different from those included in the final analysis, although baseline characteristics of the randomised population compared with the analysed population were similar, providing further reassurance.

### Conclusions and policy implications

This trial provides strong evidence for no statistically significant difference between traditional cast immobilisation and removable bracing for ankle fractures in adults. Future research should consider the importance of later stage rehabilitation after the initial immobilisation phase.

**What is already known on this topic**

Management of a fractured ankle has traditionally included cast immobilisation, which provides maximum support to bones during healing
As rigid immobilisation in a cast can result in stiff joints and weakened muscles, removable bracing might help with these problems by allowing earlier movement
It is not known which of these treatments is superior

**What this study adds**

Plaster cast immobilisation was not found to be superior to functional bracing for ankle fractures in adults at 16 weeks
Other factors will need to be considered in deciding optimal management

## Data Availability

Data sharing: Trial data are not publicly available but access to the anonymised dataset can be obtained from the corresponding author on reasonable request.

## Web extra

Extra material supplied by authors

## Notes

Contributors: RSK, MLC, MU, and ACR were conceived the study, designed the trial, obtaining grant funding, and managed the trial. RK acquired the data. NP, HP, and AH did the statistical analysis. JM and HN did the health economics analysis. All authors were responsible for the interpretation of the data and for drafting and approving the final submitted manuscript. RSK is the guarantor and accepts full responsibility for the work and the conduct of the study, had access to the data, and controlled the decision to publish. The corresponding author attests that all listed authors meet authorship criteria and that no others meeting the criteria have been omitted.

The manuscript’s guarantor (RSK) affirms that the manuscript is an honest, accurate, and transparent account of the study being reported; that no important aspects of the study have been omitted; and that any discrepancies from the study as originally planned have been explained.

Dissemination to participants and related patient and public communities: A lay summary of the results of the study will be made available to research participants on the trial website.

Provenance and peer review: Not commissioned; externally peer reviewed.

## References

[1] Court-Brown CM Caesar B . Epidemiology of adult fractures: A review. Injury 2006;37:691-7. 10.1016/j.injury.2006.04.130 16814787

[2] Kannus P Palvanen M Niemi S Parkkari J Järvinen M . Increasing number and incidence of low-trauma ankle fractures in elderly people: Finnish statistics during 1970-2000 and projections for the future. Bone 2002;31:430-3. 10.1016/S8756-3282(02)00832-3 12231418

[3] Murray AM McDonald SE Archbold P Crealey GE . Cost description of inpatient treatment for ankle fracture. Injury 2011;42:1226-9. 10.1016/j.injury.2010.08.023 20869055

[4] McPhail SM Dunstan J Canning J Haines TP . Life impact of ankle fractures: qualitative analysis of patient and clinician experiences. BMC Musculoskelet Disord 2012;13:224. 10.1186/1471-2474-13-224 23171034 PMC3517753

[5] McKeown R Kearney RS Liew ZH Ellard DR . Patient experiences of an ankle fracture and the most important factors in their recovery: a qualitative interview study. BMJ Open 2020;10:e033539. 10.1136/bmjopen-2019-033539 32024789 PMC7044932

[6] Lin CW Donkers NA Refshauge KM Beckenkamp PR Khera K Moseley AM . Rehabilitation for ankle fractures in adults. Cochrane Database Syst Rev 2012;11:CD005595. 23152232 10.1002/14651858.CD005595.pub3

[7] Kearney RS McKeown R Stevens S . Cast versus functional brace in the rehabilitation of patients treated for an ankle fracture: protocol for the UK study of ankle injury rehabilitation (AIR) multicentre randomised trial. BMJ Open 2018;8:e027242. 10.1136/bmjopen-2018-027242 30567826 PMC6303686

[8] Keene DJ Mistry D Nam J . The Ankle Injury Management (AIM) trial: a pragmatic, multicentre, equivalence randomised controlled trial and economic evaluation comparing close contact casting with open surgical reduction and internal fixation in the treatment of unstable ankle fractures in patients aged over 60 years. Health Technol Assess 2016;20:1-158. 10.3310/hta20750 27735787 PMC5075748

[9] Berntsen GK Fønnebø V Tollan A Søgaard AJ Magnus JH . Forearm bone mineral density by age in 7,620 men and women: the Tromsø study, a population-based study. Am J Epidemiol 2001;153:465-73. 10.1093/aje/153.5.465 11226978

[10] Olerud C Molander H . A scoring scale for symptom evaluation after ankle fracture. Arch Orthop Trauma Surg 1984;103:190-4. 10.1007/BF00435553 6437370

[11] Salén BA Spangfort EV Nygren AL Nordemar R . The Disability Rating Index: an instrument for the assessment of disability in clinical settings. J Clin Epidemiol 1994;47:1423-35. 10.1016/0895-4356(94)90086-8 7730851

[12] Brooks R . EuroQol: the current state of play. Health Policy 1996;37:53-72. 10.1016/0168-8510(96)00822-6 10158943

[13] Morley D Jenkinson C Doll H . The Manchester-Oxford Foot Questionnaire (MOXFQ): Development and validation of a summary index score. Bone Joint Res 2013;2:66-9. 10.1302/2046-3758.24.2000147 23673374 PMC3638305

[14] van den Berg C Haak T Weil NL Hoogendoorn JM . Functional bracing treatment for stable type B ankle fractures. Injury 2018;49:1607-11. 10.1016/j.injury.2018.06.009 29903578

[15] Kearney RS McKeown R Gallacher D . Ankle injury rehabilitation (AIR): a feasibility randomised controlled trial comparing functional bracing to plaster cast in the treatment of adult ankle fractures. Pilot Feasibility Stud 2019;5:55. 10.1186/s40814-019-0441-6 31019736 PMC6471948

[16] van Buuren G-O . mice: Multivariate Imputation by Chained Equations in R. J Stat Softw 2011;45 10.18637/jss.v045.i03.

[17] Computing RFfS. R: A language and environment for statistical computing. 2019.

[18] Dehghan N McKee MD Jenkinson RJ . Early Weightbearing and Range of Motion Versus Non-Weightbearing and Immobilization After Open Reduction and Internal Fixation of Unstable Ankle Fractures: A Randomized Controlled Trial. J Orthop Trauma 2016;30:345-52. 10.1097/BOT.0000000000000572 27045369

[19] Kortekangas T Haapasalo H Flinkkilä T . Three week versus six week immobilisation for stable Weber B type ankle fractures: randomised, multicentre, non-inferiority clinical trial. BMJ 2019;364:k5432. 10.1136/bmj.k5432 30674451 PMC6342249

