# Supplementary material for: Use of cast immobilisation versus removable brace in adults with an ankle fracture: multicentre randomised controlled trial
Source: BMJ. 2021 Jul 6;374:n1506. doi: 10.1136/bmj.n1506 (PMC8256800; doi:10.1136/bmj.n1506)
Supplement: Supplementary file 1 — Supplementary information: file 1 [file kear065381.ww1.pdf]

ANKLE INJURY REHABILITATION

ISRCTN Number:	ISRCTN15537280
Sponsor:	University of Warwick
Funding Body:	National Institute for Health Research
Ethics Approval date:	West Midlands – Edgbaston Research Ethics Committee 08/08/2017

Version Number:	6.0
Date:	01/04/2019
Stage:	Final

This protocol has regard for current HRA guidance and content

Protocol Amendments:

**Amendment No.**	**Date of Amendment**	**Date of Approval**
SA1	07/07/2017	08/08/2017
SA2	11/05/2018	30/05/2018
SA3	22/08/2018	21/09/2018
SA4	08/10/2018	11/05/2018
SA5 01/04/2019		10/05/2019

WARWICK
CLINICAL TRIALS UNIT

NHS
National Institute for Health Research

CONTACT NAMES AND NUMBERS

Role	Name, address, telephone
**Sponsor:**	Jane Prewett, Deputy Director/Head of Research Governance for Research & Impact Services,<br>University House Ground Floor Annexe, University of Warwick, Kirby Corner Road, Coventry CV4 8UW<br>Tel: 02476 522746<br>Email: sponsorship@warwick.ac.uk
**Chief Investigator:**	Rebecca Kearney, Associate Professor<br>Warwick Clinical Trials Unit, Warwick Medical School, University of Warwick Coventry, CV4 7AL<br>Tel: 02476 573156<br>Email: r.s.kearney@warwick.ac.uk
**Trial Manager:**	Rebecca McKeown<br>Warwick Clinical Trials Unit, Clinical Sciences Building, Clinical Sciences Research Laboratories, University Hospitals Coventry and Warwickshire, Clifford Bridge Road, Coventry, CV2 2DX<br>Tel: 02476 968614<br>Email: R.Mckeown.1@warwick.ac.uk
**Senior Project Manager:**	Jaclyn Brown<br>Warwick Clinical Trials Unit, Warwick Medical School, University of Warwick Coventry, CV4 7AL<br>Tel: 02476 15 0086<br>Email: J.Brown.10@warwick.ac.uk

Role	Name, address, telephone
**Co-investigators:**	Matthew Costa, Professor of Orthopaedic Trauma<br>University of Oxford, The Kadoorie Centre, John Radcliffe Hospital, Oxford, OX3 9DU<br>Tel: 01865 223114<br>Email: Matthew.costa@ndorms.ox.ac.uk
	Martin Underwood, Professor of Primary Care Research<br>Warwick Clinical Trials Unit, Warwick Medical School, University of Warwick Coventry, CV4 7AL<br>Tel: 02476 574664<br>Email: M.Underwood@warwick.ac.uk
	Nick Parsons, Principal Research Fellow<br>Warwick Medical School, University of Warwick Coventry, CV4 7AL<br>Tel: 02476 150540<br>Email: nick.parsons@warwick.ac.uk
	Anthony Redmond, Professor of Clinical Biomechanics<br>Leeds Institute of Rheumatic and Musculoskeletal Medicine<br>and Leeds NIHR Musculoskeletal Biomedical Research Unit<br>2nd Floor, Chapel Allerton Hospital, Harehills Lane,<br>Leeds, LS7 4SA<br>Tel: 0113 392 4914<br>Email: A.Redmond@leeds.ac.uk
**Statistician:**	Helen Parsons, Senior Research Fellow<br>Warwick Clinical Trials Unit, Warwick Medical School, University of Warwick Coventry, CV4 7AL<br>Tel: 02476 572665<br>Email: H.Parsons@warwick.ac.uk
**Health Economist:**	James Mason, Professor of Health Economics<br>Warwick Medical School, University of Warwick Coventry, CV4 7AL<br>Tel: 02476 151853<br>Email: J.Mason@arwick.ac.uk

Role	Name, address, telephone
**Trial Steering Committee:**	Chair:<br>Dylan Morrissey, Consultant Physiotherapist and Clinical Reader<br>Mile End Hospital<br>Bancroft Road<br>London<br>E1 4DG<br>Tel: 07941710273<br>Email: d.morrissey@qmul.ac.uk<br><br>Members:<br>Richard Grant, Lay Representative<br>Lothlorien,<br>73 Fair Isle Drive,<br>Glendale,<br>Nuneaton<br>CV10 7LL<br>Tel: 02476349789<br>Email: grantrichard73@aol.co.uk<br><br>Ben Ollivere, Clinical Associate Professor<br>Nottingham University Hospital Trust,<br>City Hospital Campus,<br>Hucknall Road,<br>Nottingham,<br>NG5 1PB<br>Tel: 07805 438317<br>Email: ben.ollivere@yahoo.co.uk<br><br>Joseph Alsousou, Academic Clinical Fellow<br>Royal Liverpool University Hospital,<br>Liverpool,<br>L69 3GA<br>Tel: 07799077922<br>Email: Joseph.alsousou@Liverpool.ac.uk

Role	Name, address, telephone
**Data Monitoring Committee:**	Chair:<br>Ed Roddy, Reader in Rheumatology<br>Research Institute for Primary Care and Health Sciences,<br>Keele University,<br>Staffordshire,<br>ST5 5BG<br>Tel: 01782 734715<br>Email: E.Roddy@keele.ac.uk<br><br>Members:<br>Elaine Nicholls, Biostatistician<br>Keele Clinical Trials Unit,<br>Keele University,<br>Staffordshire,<br>ST5 5BG<br>Tel: 01782 734750<br>Email: e.nicholls@keele.ac.uk<br><br>Michael Whitehouse, Consultant Senior Lecturer<br>Musculoskeletal Research Unit,<br>1st Floor Learning & Research Building,<br>School of Clinical Sciences,<br>Southmead Hospital,<br>Bristol,<br>BS10 5NB<br>Tel: 0117 414 7865<br>Email: michael.whitehouse@bristol.ac.uk

For general queries please contact the coordinating centre on 02476 968614 or Email: air@warwick.ac.uk

TABLE OF CONTENTS PAGE

TABLE OF CONTENTS 6

TRIAL SUMMARY 9

LIST OF ABBREVIATIONS/GLOSSARY 12

1. BACKGROUND 14

1.1 Epidemiology and burden of the condition 14

1.2 Existing knowledge 14

1.3 Hypothesis 15

1.4 Need for a trial 15

1.5 Ethical considerations 15

1.6 CONSORT 15

1.7 Assessment and management of risk 15

2. TRIAL DESIGN 16

2.1 Trial summary and flow diagram 16
2.1.1 Primary objective 19
2.1.2 Secondary objective 19
2.1.3 Outcome measures 19
2.1.4 Efficacy 20
2.1.5 Safety 20
2.1.6 Others 20

2.2 Eligibility criteria 20
2.2.1 Inclusion criteria 20
2.2.2 Exclusion criteria 21

2.3 Participant identification / Screening 21

2.4 Site Staff Training 21

2.5 Informed consent 22

2.6 Randomisation 23
2.6.1 Randomisation 23
2.6.2 Post-randomisation withdrawals, exclusions and moves out of region 24

2.7 Trial treatments / intervention 24
2.7.1 Trial treatment(s) / intervention 24
2.7.2 Compliance 25

2.8 Blinding 25
2.8.1 Methods for ensuring blinding 25

2.9 Concomitant illness and medication 25
2.9.1 Concomitant illness 25
2.9.2 Concomitant medication 25

2.10 End of trial ... 25
3. METHODS AND ASSESSMENTS ... 26
3.1 Schedule of delivery of intervention and data collection ... 26
4. ADVERSE EVENT MANAGEMENT ... 26
4.1 Definitions ... 26
4.1.1 Adverse Events (AE) ... 26
4.1.2 Serious Adverse Events (SAEs) ... 26
4.1.3 Expected Serious Adverse Events (SAEs) ... 27
4.2 Reporting AEs and SAEs ... 27
4.3 Responsibilities ... 28
4.4 Notification of deaths ... 29
4.5 Reporting urgent safety measures ... 29
5. DATA MANAGEMENT ... 29
5.1 Data collection and management ... 29
5.2 Database ... 29
5.3 Data storage ... 30
5.4 Data access and quality assurance ... 30
5.5 Data Shared with Third Parties ... 30
5.6 Archiving ... 30
6. STATISTICAL ANALYSIS ... 30
6.1 Power and sample size ... 30
6.2 Statistical analysis of efficacy and harms ... 31
6.2.1 Statistics and data analysis ... 31
6.2.2 Planned recruitment rate ... 31
6.2.3 Statistical analysis plan ... 31
6.2.3.1 Summary of baseline data and flow of participants ... 31
6.2.3.2 Primary outcome analysis ... 31
6.2.3.3 Secondary outcome analysis ... 32
6.3 Subgroup analyses ... 32
6.4 Interim analysis and criteria for the premature termination of the trial ... 32
6.5 Procedure to account for missing data ... 32
6.6 Health Economic Evaluation ... 33
7. TRIAL ORGANISATION AND OVERSIGHT ... 33
7.1 Sponsor and governance arrangements ... 33
7.2 Ethical approval ... 33
7.3 Trial Registration ... 34
7.4 Notification of serious breaches to GCP and/or trial protocol ... 34

7.5 Indemnity 34
7.6 Trial timetable and milestones 34
7.7 Administration 35
7.8 Trial Management Group (TMG) 35
7.9 Trial Steering Committee (TSC) 35
7.10 Data Monitoring Committee (DMC) 35
7.11 Essential Documentation 35
7.12 Financial Support 35
8. MONITORING, AUDIT AND INSPECTION 36
9. PATIENT AND PUBLIC INVOLVEMENT (PPI) 36
10. DISSEMINATION AND PUBLICATION 36
11. APPENDICES 37
12. REFERENCES 50

LIST OF TABLES PAGE

Table 1 Trial assessments 26

LIST OF FIGURES PAGE

Figure 1 Trial flow diagram 18

TRIAL SUMMARY

Trial Title	UK study of ankle injury rehabilitation (AIR) – multicentre randomised controlled trial	
Internal ref. number (or short title)	AIR: Ankle Injury Rehabilitation	
Clinical Phase	Phase III	
Trial Design	Multi-centre randomised controlled trial with health economic evaluation	
Trial Participants	All adults with an ankle fracture for which the treating clinician would traditionally treat the patient in a cast.	
Planned sample size	A minimum of 478 participants	
Treatment Duration	Minimum 3 weeks	
Follow-up Duration	24 Months	
Planned Trial Period	01/01/17 – 31/12/21	
	Objectives	Outcome Measures
Primary	The primary objective is to quantify and draw inferences on observed differences in the OMAS at 16 weeks between adults with an ankle fracture followed by fixed angle removable orthotic versus cast care.	OMAS
Secondary	1. To quantify and draw inferences on observed differences between the functional status (OMAS) of the trial treatment groups at 6 weeks, 10 weeks, 24 weeks and 24 months.<br>2. To draw inferences on the observed	MOXFQ<br>DRI<br>EQ-5D-5L<br>Complications<br>Resource use

	differences in ankle function assessed using the OMAS scores in operative and non-operative subgroups<br>3. To draw inferences on the observed differences in ankle function assessed using the OMAS scores in those over 50 and those under 50 years of ages subgroups<br><br>4. To quantify and draw inferences on observed differences between the functional status (MOXFQ) of the trial treatment groups at 16 weeks.<br><br>5. To quantify and draw inferences on observed differences between health related quality of life (EQ5D-5L) of the trial treatment groups at 6 weeks, 10 weeks, 16 weeks, 24 weeks, 12 months, 18 months and 24 months.<br><br>6. To quantify and draw inferences on observed differences between the functional status (DRI) of the trial treatment groups at 6 weeks, 10 weeks, 16 weeks, 24 weeks and 24 months.<br><br>7. To estimate comparative cost-utility of the two trial treatment groups at 6 weeks, 10 weeks, 16 weeks, 24 weeks 12 months, 18 months and 24 months.<br><br>8. To determine the difference in complications between the trial treatment	

	groups at 6 weeks, 10 weeks, 16 weeks, 24 weeks and 24 months.	

LIST OF ABBREVIATIONS/GLOSSARY

Abbreviation	Explanation
AE	Adverse Event
AIM	Ankle Injury Management
AIR	Ankle Injury Rehabilitation
AOUK	Arbeitsgemeinschaft für Osteosynthesefragen United Kingdom
CI	Chief Investigator
CONSORT	Consolidated Standards of Reporting Trials
CRF	Case Report Form
CTU	Clinical Trials Unit
DMC	Data Monitoring Committee
DRI	Disability Rating Index
FARO	Fixed Angle Removable Orthotic
GCP	Good Clinical Practice
HEAP	Health Economic Analysis Plan
HRA	Health Research Authority
ICF	Informed Consent Form
IRAS	Integrated Research Application System
ISRCTN	International Standard Randomised Controlled Trial Number
MCID	Minimally Clinically Important Difference
MOXFQ	Manchester-Oxford Foot Questionnaire
NIHR	National Institute for Health Research
OMAS	Olerud and Molander Ankle Score
ORIF	Open Reduction Internal Fixation
PI	Principal Investigator
PPI	Patient & Public Involvement

PROM	Patient Reported Outcome Measure
QoL	Quality of Life
RCT	Randomised Controlled Trial
REC	Research Ethics Committee
R&D	Research and Development
RfPB	Research for Patient Benefit
SAE	Serious Adverse Event
SAP	Statistical Analysis Plan
SD	Standard Deviation
SOP	Standard Operating Procedure
SPM	Senior project Manager
TMG	Trial Management Group
TSC	Trial Steering Committee
TM	Trial Manager
WCTU	Warwick Clinical Trials Unit

1. BACKGROUND

1.1 Epidemiology and burden of the condition

Ankle fractures represent 9% of the trauma workload and demand is increasing. A threefold increase is expected by 2030 due to an increase in older adults who are remaining active [1, 2]. The frequency of this injury is an increasing burden on the NHS year on year [3]. The short-term impact of this injury results in physical impairments of pain, stiffness, weakness and swelling. The longer-term impact results in prolonged time off work, development of posttraumatic arthritis and psychological consequences of depression and anxiety [4].

The main bones of the ankle are the talus (in the foot) and the tibia and fibula (in the leg). A fibrous band called the syndesmosis, which is important for ankle stability, binds the tibia and fibula. Ankle fractures that occur below the level of the syndesmosis are considered 'stable' and are usually treated with functional braces. All other ankle fractures are less stable, with some requiring open reduction and internal fixation (ORIF) with the aim of restoring stability [5].

Regardless of the decision to operate or not, the immediate management has traditionally been plaster cast immobilisation for several weeks, whilst the bone heals. A cast provides maximum support; however, there are potential problems. Firstly, there is the immediate impact on mobility for a period of around six weeks. Secondly, there are the risks associated with prolonged immobilisation: muscle atrophy, deep vein thrombosis and joint stiffness. Finally, there are the long-term consequences, which include prolonged gait abnormalities, persistent calf muscle weakness and an inability to return to previous activity levels. Alternative functional bracing may potentially address these issues [6]. However, it does not provide the same degree of support to the healing bones.

1.2 Existing knowledge

In 2010 an orthopaedic trauma network (AOUK) undertook a research priority exercise [7]. One of the top priority questions was to establish if there is a clinical advantage to different management plans following an ankle fracture.

A Cochrane Review addressed this topic in 2012 [8]. It concluded that immediate management involving the use of a removable brace, therefore allowing early movement, might reduce activity limitation, pain and improve ankle movement compared to a non removable cast. However, these potential advantages needed to be balanced against the increased incidence of adverse events. Consequently, future research was recommended to confirm the clinical and cost effectiveness of bracing following an ankle fracture [8].

The authors of the Cochrane review found no ongoing studies that will provide definitive research on this question. Using the same search strategy, one additional ongoing trial has been identified (ISRCTN15497399). This is a randomised controlled trial taking part in three centres, with narrow eligibility criteria (only operative ankle fractures) and a primary end point of seven weeks post injury. One recently completed RCT has also been identified as an abstract in a conference publication. This study was completed in Canada across two trauma centres, including 110 participants. The authors reported increased function in the functional bracing group at six weeks. This study adds further evidence as to the potential benefits of early exercise, however the generalisability to a UK setting is questionable and their primary outcome measure was a non-validated measure of function (return to work). Therefore, these results do not provide definitive evidence and the need for a trial is still clear.

The CI (Rebecca Kearney) has subsequently led a feasibility RCT funded by NIHR RfPB comparing cast with fixed angle removable orthotic (FARO) for the management of operative and non-operative ankle fractures. The trial ran from August 2015 and completed May 2017, successfully recruiting 50 participants, at a rate of five participants per month and achieving more than 80% follow up at each time point over a six month time period.

Informed by the data collected from the AIR feasibility trial, the team have subsequently designed the main study, also funded by NIHR. The main changes from the feasibility design have included earlier follow up time points, change in primary outcome measure from the Manchester Oxford Foot Questionnaire (MOXFQ) to Olerud and Molander Ankle Score (OMAS), eligibility criteria and refinement of data collection forms. The protocol paper has been published [9] and the subsequent main publication detailing these findings further is in the process of submission.

1.3 Hypothesis

Research Question:
In adults with an ankle fracture suitable for cast immobilisation, does a fixed angle removable orthotic (FARO) improve OMAS 16 weeks post randomisation when compared to cast immobilisation?

Null Hypothesis:
There is no difference in the OMAS at 16 weeks post randomisation between adults with an ankle fracture managed with FARO versus cast immobilisation.

1.4 Need for a trial

With the incidence of ankle fractures on the rise, and in light of the large personal and societal cost associated with the injury, this gap in the evidence is a clear priority. Therefore, now is the right time to conduct a trial of FARO versus cast for patients with an ankle fracture.

1.5 Ethical considerations

The trial will be conducted in full conformance with the principles of the Declaration of Helsinki and to Good Clinical Practice (GCP) guidelines. It will also comply with all applicable UK legislation and Warwick Standard Operating Procedures (SOPs). All data will be stored securely and held in accordance with Data Protection Act 1998.

1.6 CONSORT

The trial will be reported in line with the CONSORT (*Con*solidated *S*tandards *o*f *R*eporting *T*rials) statement.

1.7 Assessment and management of risk

Both FARO and cast immobilisation are currently used across the NHS for the management of ankle fractures. Consequently, both trial interventions reflect current standard practice and do not expose trial participants to any substantial risks over and above standard care currently received.

2. TRIAL DESIGN

2.1 Trial summary and flow diagram

This is a UK multi-centre, randomised controlled trial. All adults with a fractured ankle under the care of a clinician at any of the named recruiting sites are potentially eligible. New patients with an ankle fracture are reviewed each day by the trauma team either:

1. As an inpatient admission on the trauma ward.

2. As an outpatient in fracture clinic.

Following identification of a potential participant with a new ankle fracture by the trauma team a suitably trained member of the research team at each site will be contacted to undertake eligibility checks in conjunction with the trauma team.

All adults with an ankle fracture for which the treating clinician would consider cast a reasonable management option will be assessed for study entry. All potential participants meeting the entry criteria will be checked for eligibility and details entered on the monthly screening log. All eligible potential participants who are willing to be approached by a suitably trained member of the research team will be provided with verbal and written information about the study. At which point they will follow one of two pathways:

1. Deemed appropriate for surgery and placed/waiting to be placed on a suitable trauma list to undergo surgery. Following surgery, they will be managed as per normal clinical practice at each participating site to allow the wound to heal and discharged home. They will then return to an outpatient clinic to check the wound is healing satisfactory by the trauma team (usually second week post op). At this time if the wound assessment is satisfactory a suitably trained member of the research team will re-approach the participant to confirm eligibility with the trauma team.

2. Deemed not appropriate for surgery. In these cases the suitably trained member of the research team will approach the participant to confirm eligibility with the trauma team.

If a potential participant is deemed eligible and is willing to take part in the study, a suitably trained member of the research team will then be responsible for completing consent procedures, baseline demographic data and pre and post injury functional outcomes using OMAS, DRI and EQ-5D-5L before randomisation. The participant will then be randomised using a web based randomisation service to either the FARO intervention or the cast control, on a 1:1 basis, stratified by centre, age and operative/non-operative management.

Four hundred and seventy eight participants will be randomised in total, across participating centres. Both interventions are currently used in the NHS, consequently delivery of the randomised allocation will follow normal practice. The participants will then attend follow up appointments as per routine clinical practice. All trial related follow up will be completed through postal follow up centrally from Warwick CTU at six weeks, 10 weeks, 16 weeks, 24 weeks, 12 months, 18 months and 24 months post randomisation. At each follow up (except 12 and 18 months) complications, functional status (OMAS and DRI), health related quality of life (EQ-5D-5L) and resource use questionnaires will be collected. For the 12 and 18 month data point only EQ-5D-5L and resource use will be collected. Sixteen weeks will be the primary end point and also include functional status measured by MOXFQ.

The local principal investigator and research team at each site cannot be blind to treatment as they will be delivering the interventions. None of these team members will have a role in the collection of follow up participant data beyond reporting serious adverse events.

A trial management group (TMG), trial steering committee (TSC) and data monitoring and ethics committee (DMC) will oversee the trial.

Figure 1 Trial flow diagram

1. All adults with a closed ankle fracture for which the treating clinician would consider plaster cast a reasonable management option.

2. Within 3 weeks of operative management or injury if non-operative.

Eligible and consenting participants complete baseline CRFs

Randomised
(minimum n = 478)

Plaster cast
(n=239)

Postal follow up:

6 weeks

10 weeks

16 weeks

24 weeks

12 months

18 months

24 months

Functional bracing
(n = 239)

Postal follow up:

6 weeks

10 weeks

16 weeks

24 weeks

12 months

18 months

24 months

__**Exclusion criteria:**__

1. Ankle fracture secondary to known metastatic disease.

2. Complex intra-articular fracture (e.g. Pilon fracture)

3. In the opinion of the surgeon the patient would require close contact casting.

4. In the opinion of the surgeon the patient would require manipulation and moulded cast.

5. Wound complications contraindicating FARO intervention.

6. Previous ankle fracture randomised in the present trial.

7. Evidence that the patient would be unable to adhere to trial procedures or complete postal questionnaires.

8. Known pre-existing neuropathic joint disease contraindicating FARO intervention

Aims and objectives

2.1.1 Primary objective

Primary Objective:
To quantify and draw inferences on observed differences in the OMAS between FARO and cast treatment groups at sixteen weeks after randomisation.

2.1.2 Secondary objective

Secondary Objectives:

1) To quantify and draw inferences on observed differences in ankle function assessed using the OMAS score at 6 weeks, 10 weeks, 24 weeks and 24 months and MOXFQ 16 weeks after randomisation.

2) To quantify and draw inferences on observed differences in health related quality of life (EQ5D-5L) between trial treatment groups at 6 weeks, 10 weeks, 16 weeks, 24 weeks, 12 months, 18 months and 24 months after randomisation.

2) To quantify and draw inferences on observed differences in disability rating (DRI) between trial treatment groups at 6 weeks, 10 weeks, 16 weeks, 24 weeks and 24 months after randomisation.

3) To quantify and draw inferences on observed differences on complication rates between trial treatment groups at 6 weeks, 10 weeks, 16 weeks, 24 weeks and 24 months after randomisation.

4) To estimate comparative cost-utility of the two trial treatment groups and collect resource use data at 6 weeks, 10 weeks, 16 weeks, 24 weeks, 12 months, 18 months and 24 months after randomisation.

2.1.3 Outcome measures

- Primary:

Olerud and Molander Ankle Score (OMAS): Is a self-administered questionnaire. It is a suitable outcome tool for assessing symptoms after an ankle fracture. The score is based on nine different items: pain, stiffness, swelling, stair climbing, running, jumping, squatting, supports and work/activities of daily living [10]. The scoring system correlates well with parameters considered to summarise the results after this type of injury.

- Secondary:

EQ-5D-5L: Is a validated, generic health-related quality of life measure consisting of five dimensions each with a 5-level answer possibility. Each combination of answers can be converted into a health utility score. It has good test-retest reliability, is simple for participants to use, and gives a single preference-based index value for health status that can be used for broader cost-effectiveness comparative purposes [11].

MOXFQ: Is a validated questionnaire which is self-reported (filled in by the participant). It contains 16 items, each with 5 response options comprising 3 separate underlying dimensions: Walking/standing problems (7 items), foot pain (5 items) and issues related to social interaction (4 items). Item responses are each scored from 0 to 4, with 4 representing the most severe state. The scale scores representing each dimension are produced by summing the responses to each item within that dimension. Raw scale scores are then converted to a metric (0-100; 100=most severe).

DRI: Is a self-administered questionnaire. It consists of 12 items specifically related to function of the lower limb. The DRI has been proven to be a practical clinical and research instrument, with good responsiveness and acceptability for assessment of disability caused by impairment in the lower limb [12].

Complications: All complications will be recorded, including mal-union, delayed/non-union, infection, wound complications, vascular injury, neurological injury, and venous thromboembolism. A record will also be kept of any other surgery required in relation to the index fracture. All baseline X-Ray/radiographs and also the last X-Ray/radiograph taken before the primary outcome point of 16 weeks will be collected.

Resource use questionnaires: The primary analysis will concentrate on direct intervention and healthcare/personal social services costs, while wider impact (societal) costs will be included within the sensitivity analyses. Relevant resource use questionnaires will be administered to participants at baseline and all follow-up points, to collect resource use data associated with the interventions under examination.

2.1.4 Efficacy

Primary: OMAS

Secondary: DRI, MOXFQ

All outcome measures will be paper based and collected at baseline (pre randomisation) face to face in the clinical setting. Subsequent data will be collected by postal questionnaire sent from Warwick CTU (WCTU) at six weeks, 10 weeks, 16 weeks, 24 weeks and 24 months post randomisation. MOXFQ will be collected at 16 weeks only.

2.1.5 Safety

Adverse event data

At each data point participants will be asked by postal questionnaire sent from WCTU if they have had any adverse events and how they were managed. Additionally, PIs will be asked to comply with procedures for reporting Serious Adverse Events (SAEs) to the AIR central office within 24 hours of becoming aware of an event.

2.1.6 Others

Health economics

All outcome measures will be paper based and collected at six weeks, 10 weeks, 16 weeks, 24 weeks, 12 months, 18 months and 24 months post randomisation by postal questionnaire sent from WCTU.

2.2 Eligibility criteria

Eligible to be included in the trial if potential participants meet the following criteria:

2.2.1 Inclusion criteria

1. Provision of written informed consent

2. Aged 18 years or over

3. A closed ankle fracture for which the treating clinician would consider plaster cast a reasonable management option.

4. Within 3 weeks of operative management or injury if non-operative.

2.2.2 Exclusion criteria

1. Ankle fracture secondary to known metastatic disease.

2. Complex intra-articular fracture (e.g. Pilon fracture)

3. In the opinion of the surgeon the patient would require manipulation and close contact casting.

4. In the opinion of the surgeon the patient would require manipulation and moulded cast.

5. Wound complication that would contraindicate fixed angle removable orthotic intervention.

6. Previous ankle fracture randomised in the present trial.

7. Evidence that the patient would be unable to adhere to trial procedures or complete postal questionnaires.

8. Known pre-existing neuropathic joint disease contraindicating FARO intervention

2.3 Participant identification / Screening

New patients with an ankle fracture are reviewed each day by the trauma team either as inpatient admissions on the trauma ward or as an outpatient in fracture clinic. Following identification of a potential participant with a new ankle fracture by the trauma team a suitably trained member of the research team at each site will be contacted to undertake paper eligibility checks in conjunction with the trauma team. The research team member will complete the eligibility checklist and sign to confirm that eligibility checks were made in conjunction with and have been verified by a suitably qualified member of the trauma team. The trauma team member will then document this on the patient file, a quality assurance plan to check this process will be detailed further in the monitoring plan held at WCTU.

All people meeting the entry criteria will be checked for eligibility and entered on the monthly screening log. All eligible potential participants who are willing to be approached by a suitably trained member of the research team will be provided with verbal and written information about the study. If potential participants do not undergo surgery they will be then be asked if they wish to take part in the study. All new non-operatively managed potential participants will have a maximum of 21 days from injury to make a final decision.

If potential participants do undergo surgery, the suitably trained member of the research team will re-approach the participant for consent when they return for a wound check (usually 14 days, up to maximum of 21 days), at an outpatient appointment.

To ensure all members of the trauma team are informed of the study, and to raise the profile of the study, where possible, publicity posters will be placed in clinical areas.

2.4 Site Staff Training

An appropriately trained member of the research team will be responsible for confirming eligibility with trauma teams, obtaining consent, collecting baseline data and subsequent SAE reporting. The

Trial Manager will provide training to complete these tasks via a presentation outlining an overview of the trial (key personnel, protocol, management and oversight) and clinical reporting form (CRF) completion, such as SAE reporting, withdrawals, screening logs and data clarifications. This training will take place prior to recruitment with the local PI and research team who will carry out trial related procedures.

This local team will then be responsible for distribution of the training materials across the wider trauma team using opportunities to present during clinical training and departmental meetings on an ad hoc basis. This wider training will occur throughout the recruitment phase due to the nature of rotating and changing trauma staff members.

When a new research team member or PI is appointed at local sites the Trial Manager will be responsible for re-distributing trial-training materials and ensuring delegation logs are updated.

No specific training on administering trial interventions is required as they are used as part of routine clinical practice for a range of trauma and orthopaedic presentations.

2.5 Informed consent

Consent materials:

Potential participants will be provided with verbal and written information about the study. A list of information the research team should cover before consent is obtained will be provided to ensure that all essential information is discussed with the potential participant.

Timing of consent:

Written informed consent will be obtained by a suitably trained member of the research team at each site as per the delegation log, after allowing sufficient time for the potential participant to consider their decision and ask questions about the trial. Sufficient time for some potential participants may result in a decision to take part in the trial immediately after receiving all relevant information; this is reflective of clinical practice. Alternatively, if potential participants would like to leave the clinic with the information and make a decision at a later date of their choosing, they will be free to do so within the time limits specified in the section entitled ‘intervention’. The definition of ‘sufficient time’ is at the discretion of the potential participant being approached to take part.

Consent responsibility:

The PI retains overall responsibility for informed consent at their site and will ensure that any person delegated responsibility to participate in the informed consent process is duly authorised, trained, qualified and competent.

New information:

Any new information that arises during the trial that may affect participants’ willingness to take part will be reviewed by the TSC; if necessary this will be communicated to all participants. A revised consent form will be completed if necessary.

Participants GP will be informed by letter that they are taking part in this clinical trial. Participants may decline their GP being informed of their participation in the trial involvement by not initialling the appropriate box on the consent form.

Decline/withdrawal:

The right of a potential participant to refuse participation without giving reasons will be respected, and recorded on the screening log. The participant will remain free to withdraw at any time without giving reasons and without prejudice to any further treatment, and will be provided with a contact point where he/she may obtain further information about the trial.

2.6 Randomisation

2.6.1 Randomisation

Pre-randomisation eligibility checks will be carried out to ensure that potential participants meet the eligibility criteria and are not randomised in error. Written informed consent for entry into the trial and baseline assessment must be obtained prior to randomisation. Subjects will be randomised strictly sequentially, as they become registered as eligible for randomisation on the web based system. Allocation concealment will be maintained by an independent randomisation team who will be responsible for generation of the sequence and will have no role in the allocation of participants.

The treatment group will be allocated by computer using a minimisation algorithm with a random element and stratification by centre, age and operative/non operative management following use of a secure web based randomisation service. Minimisation is a better option than conventional stratification with variable block sizes, due to the relatively small number of participants expected in some strata. The randomisation service will be available 24 hours a day, seven days a week to facilitate the inclusion of all eligible participants. The randomisation system will allocate each participant a unique trial number. A confirmation email will be automatically generated to the research site containing the randomisation details. A member of the research team will inform the relevant trauma team member who routinely administers the trial interventions. The trauma team member will then administer the treatment allocation.

Stratification by centre will help ensure that any clustering effect related to the centre itself will be equally distributed in the trial arms. Stratification on the basis of age will be used to ensure that younger participants with normal bone quality sustaining high-energy fractures and older participants with low-energy (fragility) fractures related to osteoporosis are also balanced between arms. The age group stratification will also be used as a proxy for bone density.

In a large study in Norway involving 7600 participants, found that bone mineral density remains stable up until the age of 50 years. After the age of 50, bone mineral density decreased steadily in males, whilst in females there was an initial decline between the ages of 50 and 65, with a further decline in the age groups thereafter [13]. A recent study by Court-Brown [1], assessed over 1000 patients with a fracture. This study confirmed that there is a clear bimodal distribution according to the age of the patient. The crossover of the two peaks of incidence was around 50 years of age. These studies provide strong evidence that patients over the age of 50 become increasingly vulnerable to fragility fractures. Based on this information; and to align with recently published NIHR multi centre RCTs on ankle fracture management [14], we have chosen an age of 50 as the stratification cut-off for this trial.

A final stratification based on operative/non operative presentation will be implemented. This will be used to estimate the extent of the ligamentous injury and the stability of the joint. It will ensure that any effect related to severity of injury is equally distributed amongst the trial arms.

2.6.2 Post-randomisation withdrawals, exclusions and moves out of region

Withdrawal:

Participants may be discontinued from the trial treatment and/or the trial at any time without prejudice. Unless a participant explicitly withdraws their consent, they should be followed-up wherever possible and data collected as per the protocol until the end of the trial. For participants explicitly withdrawing consent for follow up procedures, trial data obtained up until the point of withdrawal will be included in the final analysis of the study. Participants will have the option to withdraw from the trial-related questionnaires, but continue to provide routine NHS data for the purposes of the trial e.g. hospital records of subsequent treatment for the ankle fracture.

Participants who withdraw will not be replaced in the trial and a corresponding withdrawal CRF will be completed by the Trial Manager.

Participants may be withdrawn from the trial at the discretion of the investigator and/or Trial Steering Committee due to safety concerns.

Follow up:

Outcome questionnaires can be completed over the telephone, if postal copies are not returned. Text messages will be sent to participants to inform them that a questionnaire is due or on its way. Text messages will only be sent to those participants who have given their prior consent to this by initialling the corresponding box on the consent form and providing their mobile telephone number. Details pertaining to the timing and nature of planned follow up procedure for telephone/post/text message are further detailed in the data management plan.

2.7 Trial treatments / intervention

2.7.1 Trial treatment(s) / intervention

All participants who require ankle fixation will have this performed according to the preferred technique of the operating surgeon. The details of the surgery will be left to the discretion of the surgeon to ensure that the results of the trial can be generalised. However, a suitably trained member of the research team will complete details of the procedure undertaken within the baseline CRF. All participants will then receive normal local care until satisfactory clinical wound check (usually two weeks post operatively, up to maximum three weeks), at which point the intervention will be applied by a suitably trained member of the trauma team.

All participants not receiving surgery will be approached to take part in the trial on first presentation to the trauma team fracture clinic, and will be eligible for trial participation up to a maximum of 3 weeks from injury.

Weight bearing status will be at the discretion of the treating member of the trauma team and recorded on subsequent CRFs.

Control Group - Standard Plaster Cast:

All participants in the control arm will be fitted with cast immobilisation for a minimum of three weeks. It is expected that the control intervention will not exceed eight weeks, however this will be recorded and monitored by the TMG.

Intervention Group – Functional Bracing:

All participants in the intervention arm will be fitted with a FARO for a minimum of three weeks. It is expected that the intervention will not exceed eight weeks, however this will be recorded and monitored by the TMG. Throughout this period participants will be encouraged to remove their functional brace to complete active unloaded ankle range of movement exercises. An information sheet explaining these exercises will be handed out to all participants randomised to this trial arm.

Rehabilitation:

Following the trial intervention period, in this pragmatic trial, any other rehabilitation input (including a formal referral to physiotherapy) will be left to the discretion of the treating trauma team member. However, a record of any additional rehabilitation, together with a record of any other interventions will be recorded on follow up CRFs.

2.7.2 Compliance

Quality assurance checks will be carried out via CRF completion to assess compliance with the above intervention delivery. Any deviations noted from the outlined trial interventions will be monitored by the TMG. If required further training will be implemented to resolve any inconsistencies.

2.8 Blinding

2.8.1 Methods for ensuring blinding

As the type of rehabilitation used is clearly visible, the participants cannot be blind to their treatment. In addition, the treating clinical team cannot be blind to the treatment, but will take no part in the assessment of potential participants.

The questionnaire data will be collected and entered onto the trial central database via phone and postal mechanisms by a suitably trained member of the research team for all data points. The inputting member of the research team cannot be blinded to the trial interventions, however data will be presented to the DMC using a combination of open and closed reports that will be further detailed in the SAP.

2.9 Concomitant illness and medication

2.9.1 Concomitant illness

Details of any concomitant illness will be recorded at trial entry.

2.9.2 Concomitant medication

Details of concomitant medication will be recorded at trial entry, as detailed in the baseline CRF.

2.10 End of trial

The trial will end when all participants have completed their 24 month follow-up.

The trial will be stopped prematurely if:

- Mandated by the Ethics Committee
- Following recommendations from the Data Monitoring Committee (DMC)
- Funding for the trial ceases

The Research Ethics Committee will be notified in writing within 90 days when the trial has been concluded or within 15 days if terminated early.

3. METHODS AND ASSESSMENTS

3.1 Schedule of delivery of intervention and data collection

Table 1 Trial assessments

**Visit**	**1**	**2**	**3**	**4**	**5**	**6**	**7**	**8**	**9**
**Visit Window** (No. Weeks ± No. Days)	**Pre - Consent**	**Baseline**	**6wk (± 2wk) After V2**	**10wk (± 2wk) After V2**	**16 wk (± 4 wk) After V2**	**24 wk (± 4 wk) After V2**	**12 m (± 1m) After V2**	**18 m (± 1m) After V2**	**24 m (± 1m) After V2**
Eligibility Check	✓								
Written and verbal information provided	✓								
Written informed consent		✓							
Baseline CRFs (Pre and Post injury)		✓							
Randomisation		✓							
Intervention delivery		✓							
OMAS		✓	✓	✓	✓	✓			✓
MOXFQ		✓			✓				
DRI		✓	✓	✓	✓	✓			✓
EQ5D5L		✓	✓	✓	✓	✓	✓	✓	✓
Resource use questionnaires			✓	✓	✓	✓	✓	✓	✓
Complications			✓	✓	✓	✓			✓

4. ADVERSE EVENT MANAGEMENT

4.1 Definitions

4.1.1 Adverse Events (AE)

An Adverse Event (AE) is defined as any untoward medical occurrence in a participant which does not necessarily have a causal relationship with this treatment/intervention. AEs related to the management of an ankle fracture will be recorded on the appropriate CRF for routine return to the AIR central office and reported to the relevant oversight committees.

4.1.2 Serious Adverse Events (SAEs)

An AE is considered a SAE if it is an untoward medical occurrence that fulfils one or more of the following criteria:

- Results in death
- Is immediately life-threatening
- Requires hospitalisation or prolongation of existing hospitalisation
- Results in persistent or significant disability or incapacity
- Is a congenital abnormality or birth defect

- Is an important medical condition.

SAEs that are determined by the local PI to have no causal relationship with the intervention will not be required for onward reporting to relevant oversight committees.

4.1.3 Expected Serious Adverse Events (SAEs)

SAEs that may be expected as part of the interventions are: complications of anaesthesia or surgery (e.g. wound complications, infections, damage to a nerve or blood vessel and thromboembolic events) and secondary operations for or to prevent infection, malunion, non-union or for symptoms related to the metalwork. These will be recorded on the participant's CRF for routine return to the AIR central office and reported to the relevant oversight committees. All participants experiencing SAEs will be followed-up as per protocol until the end of the 2 year follow up period.

4.2 Reporting AEs and SAEs

All AEs and SAEs will be entered onto the appropriate reporting form and reported to WCTU using a dedicated AIR and QA resource account within 24 hours of the investigator becoming aware of them. Once received, causality and expectedness will be confirmed by the Chief Investigator. SAEs that are deemed to be unexpected and possibly, probably or definitely related to the trial interventions will be notified to the Research Ethics Committee (REC) within 15 days. All such events will be reported to the TMG at their next meeting. All SAEs that occur between the date of consent and the end of two year follow up for the participant will be reported. For each SAE the following information will be collected:

- full details in medical terms and case description
- event duration (start and end dates, if applicable)
- action taken
- outcome
- seriousness criteria
- causality (i.e. relatedness to intervention), in the opinion of the investigator
- whether the event would be considered expected or unexpected.

**Relationship to trial intervention**	**Description**
Unrelated	There is no evidence of any causal relationship
Unlikely to be related	There is little evidence to suggest there is a causal relationship (e.g. the event did not occur within a reasonable time after administration of the trial intervention or device). There is another reasonable explanation for the event (e.g. the patient's clinical condition, other concomitant treatment).
Possible relationship	There is some evidence to suggest a causal relationship (e.g. because the event occurs within a reasonable time after administration of the trial intervention or device). However, the influence of other factors may have contributed to the event (e.g. the patient's clinical condition, other concomitant treatments).

Probable relationship	There is evidence to suggest a causal relationship and the influence of other factors is unlikely.
Definitely related	There is clear evidence to suggest a causal relationship and other possible contributing factors can be ruled out.

Once received by the AIR trial office all AEs and SAEs will be placed in one of two categories for onward reporting to oversight committees:

1. Local event (e.g. fracture non-union).

2. Systemic event (e.g. deep vein thrombosis).

4.3 Responsibilities

Principal Investigator (PI):

Checking for SAEs and AEs when participants attend for treatment / follow-up.

1. Using clinical judgement in assigning seriousness, causality and expectedness
2. Ensuring that all SAEs are recorded and reported to the Sponsor within 24 hours of becoming aware of the event and provide further follow-up information as soon as available.
3. Ensuring that AEs are recorded.

Chief Investigator (CI) / delegate or independent clinical reviewer:

1. Clinical oversight of the safety of patients participating in the trial, including an ongoing review of the risk / benefit.
2. Using clinical judgement in confirming seriousness, causality and expectedness of SAEs.
3. Using clinical judgement in assigning expectedness.
4. Immediate review of all related and unexpected SAEs
5. Ongoing review of specific SAEs in accordance with the trial risk assessment and protocol as detailed in the Trial Monitoring Plan.
6. Production and submission of annual reports to the relevant REC.

Sponsor:

1. Central data collection and verification of SAEs, according to the trial protocol.
2. Reporting safety information to the CI, delegate or independent clinical reviewer for the ongoing assessment of the risk / benefit according to the Trial Monitoring Plan.
3. Reporting safety information to the independent oversight committees identified for the trial (Data Monitoring Committee (DMC) and / or Trial Steering Committee (TSC)) according to the Trial Monitoring Plan.
4. Expedited reporting of related and unexpected SAEs to the REC within required timelines.
5. Notifying Investigators of related and unexpected SAEs that occur within the trial.

Trial Steering Committee (TSC):

In accordance with the Trial Terms of Reference for the TSC, periodically reviewing safety data and liaising with the DMC regarding safety issues.

Data Monitoring Committee (DMC):

In accordance with the Trial Terms of Reference for the DMC, periodically reviewing unblinded overall safety data to determine patterns and trends of events, or to identify safety issues, which would not be apparent on an individual case basis.

4.4 Notification of deaths

Only deaths that are assessed to be caused by the intervention will be reported to the sponsor. This report will be immediate.

4.5 Reporting urgent safety measures

If any urgent safety measures are taken the CI/Sponsor shall immediately and in any event no later than 3 days from the date the measures are taken, give written notice to the relevant REC of the measures taken and the circumstances giving rise to those measures.

5. DATA MANAGEMENT

Personal data collected during the trial will be handled and stored in accordance with the 1998 Data Protection Act.

The Case Report Forms will be designed by the TM in conjunction with the TMG. All electronic patient-identifiable information will be held on a secure, password-protected database accessible only to authorised personnel. Paper forms with patient-identifiable information will be held in secure, locked filing cabinets within a restricted area of Warwick Medical School. All subsequent CRFs will identify participants by a trial number only. Direct access to source data/documents will be required for trial related monitoring. All paper and electronic data will be retained for at least ten years after completion of the trial.

If any confidential information is disclosed by a participant indicating an issue which may jeopardise the safety of the participant or another person, such information will be brought to the attention of the CI and their treating clinical team as appropriate so an appropriate course of action can be determined.

5.1 Data collection and management

The CRFs will be developed to collect all required trial data. Suitably trained members of the research team will then complete and return CRFs to the AIR trial office. The AIR team will check and enter the data onto the trial database following instructions set out in the data management plan and in accordance with WCTU SOPs.

Suitably trained members of the research team will be required to complete CRFs at baseline and any occurring SAEs on an ongoing basis. All other outcomes/CRFs will be collected directly from the trial participants by postal mechanisms at the follow up time points. Participants will receive a £5 voucher incentive within the 16 week follow up questionnaire to aid retention and return. Procedures for chasing missing data/unreturned questionnaires are outlined in the data management plan.

5.2 Database

The database will be developed by the Programming Team at WCTU and all specifications (i.e. database variables, validation checks, screens) will be agreed between the programmers and appropriate trial staff.

5.3 Data storage

All essential documentation and trial records will be stored by WCTU in conformance with the applicable regulatory requirements and Warwick SOPs. Access to stored information will be restricted to authorised personnel only.

5.4 Data access and quality assurance

Personal data collected during the trial will be handled and stored in accordance with the 1998 Data Protection Act. All electronic patient-identifiable information will be held on a secure, password-protected database accessible only to authorised personnel. Paper forms with patient-identifiable information will be held in secure, locked filing cabinets within a restricted area of Warwick Medical School. All subsequent CRFs following baseline, will identify participants by a trial number only. Direct access to source data/documents will be required for trial related monitoring. All paper and electronic data will be retained for at least ten years after completion of the trial.

All names and addresses of trial participants will not be disclosed to anyone other than relevant members of the trial team. The investigator must arrange for retention of trial records on site in accordance with GCP. Direct access to source data/documents will be required for trial-related monitoring or audit by WCTU, internal audit, regulatory authorities and ethics committees.

Access to the final data set will be limited to authorised personnel at Warwick CTU and co-investigators, subject to local data sharing agreements.

5.5 Data Shared with Third Parties

Any application received by third parties will be discussed and approval sought by the TMG.
Relevant data sharing agreements would be put in place.

5.6 Archiving

Trial documentation and data will be archived for at least ten years after completion of the trial, in keeping with WCTU SOPs.

6. STATISTICAL ANALYSIS

6.1 Power and sample size

The primary outcome for this study is the OMAS 16 weeks post injury. The OMAS is measured on a scale between 0 and 100, where higher scores denote better function. The minimum clinically important difference (MCID), or smallest between group difference that is likely to be clinically meaningful beyond measurement error for foot and ankle conditions is a change of 10 points. This is consistent with the AIM study [14], which set the OMAS equivalence margin between groups to be 6 points. It is also consistent with other similar outcome measures such as the Foot and Ankle Outcome Score [15], and visual analogue pain scores in acute injury; that report MCIDs of approximately 10 points on a 100 point scale.

The standard deviation (SD) of the OMAS at six months after injury from previous feasibility work was approximately 28 points. To account for any variation arising from recruiting from multiple study centres and to allow that the primary outcome has been moved from this time point, we have selected a conservative estimation of the trial SD of 30 points. This corresponds to a moderate standardised effect size of 0.33. Hence, the total trial sample size required to detect a difference of

10 points given a SD of 30 points with two-sided significance set at 5% and 90% power is a minimum of 382 participants.

Allowing a margin of 20% loss during follow-up (whilst striving to keep this below 10%), this gives a figure of 478 participants in total. Therefore, a minimum of 239 participants randomised to each group will provide 90% power to detect a difference of 10 points in OMAS at sixteen weeks at the 5% level. If possible, recruiting a larger sample would enable the between group differences of the two groups to be estimated with higher precision. The minimum 382 participants would create a 95% CI of width 8.5 points. If, for example, 625 participants were recruited, this would yield data on around 500 participants at 20% loss to follow up and would enable a 95% confidence interval (CI) of width 7.4 points to be constructed.

6.2 Statistical analysis of efficacy and harms

6.2.1 Statistics and data analysis

Unless otherwise stated further detail in relation to the subsection of 6.2 will be detailed in a statistical analysis plan (SAP), which will be agreed with the Data Management Committee (DMC). No interim analyses are planned, and will be performed only where directed by the DMC. Furthermore, all primary analyses are planned to be on an intention to treat basis with secondary per protocol analysis.

6.2.2 Planned recruitment rate

Recruitment from centres will be based on a staged roll out. The expected rate of recruitment is based on a combination of feasibility data and audit data from the lead centre. The average number of potential participants eligible for the feasibility has been approximately 8.5 per month, recruiting on average five per month. The other centres involved in the trial will all be trauma centres with similar catchment areas to the lead centre. Experience from previous multi-centre trials has shown that recruitment outside of the lead centre tends to occur at a lower rate. As such, a conservative recruitment rate of three participants per month per centre is estimated. We intend to recruit from approximately 18 centres over approximately 17 months.

6.2.3 Statistical analysis plan

All data will be analysed and reported in accordance with the CONSORT statement. Treatment effects will be presented, with appropriate 95% confidence intervals, for both the unadjusted and adjusted analyses. Tests will be two-sided and considered to provide evidence for a significant difference if p-values are less than 0.05 (5% significance level). All analyses will be conducted as intention to treat unless otherwise specified.

6.2.3.1 Summary of baseline data and flow of participants

Baseline data will be summarised to check comparability between treatment arms, and screening data will be checked to highlight any characteristic differences between those individuals in the study, those ineligible, and those eligible but withholding consent. A CONSORT chart illustrating participant flow throughout the study will also be produced. Standard statistical summaries will be presented for the primary outcome measure (OMAS) and all secondary outcome measures.

6.2.3.2 Primary outcome analysis

The main analysis will investigate differences in the primary outcome measure, sixteen weeks after randomisation, between the two treatment groups. Unadjusted and adjusted regression analyses will be used to estimate the between group difference. The adjusted analyses will adjust for the stratification variables, baseline scores and any other clinically important variables. More specifically, adjusted mixed-effects modelling will be used where the recruiting centre will be included as a random effect to allow for possible heterogeneity in patient outcomes due to the

recruiting centre. Since individual clinicians will treat only a small number participants enrolled in the trial, we do not expect clinician specific effects to be important in this study and hence will not be modelled. This adjusted mixed-effects linear regression analysis will be reported as the primary analysis, and will be used to assess evidence for differences in outcomes between intervention arms.

6.2.3.3 Secondary outcome analysis

Descriptive statistics of patient reported outcome measure (PROM) data (i.e. OMAS, MOXFQ, EQ5D and DRI) at each time point will be constructed with between group analyses following the method set out for the primary analysis above. Patterns of recovery will also be explored.

Complications will be summarised with between groups comparisons evaluated using chi-squared tests. Temporal patterns of any complications will be presented graphically and if appropriate, a time-to-event analysis (e.g. Kaplan-Meier survival analysis) will be used to assess the overall risk and risk within individual classes of important complications (e.g. non-union).

6.3 Subgroup analyses

Two pre-specified sub-group analyses will be undertaken to assess whether there is evidence that the intervention effect differs between whether:

• The study participants receives operative or non-operative treatment prior to the study intervention

• The study participants are aged 60 or over at study randomisation

The subgroup analyses will follow the methods described for the primary analysis, with additional interaction terms incorporated into the mixed-effects regression model to assess the level of support for these hypotheses.

The study is not powered to formally test these hypotheses, so they will be reported as exploratory analyses only, and as subsidiary to the analysis reporting the main effects of the intervention in the full study population.

6.4 Interim analysis and criteria for the premature termination of the trial

A detailed statistical analysis plan (SAP) will be agreed with the Data Management Committee (DMC) at the start of the study. No interim analyses are planned and interim analyses will be performed only where directed by the DMC, and with the agreement of the TSC.

6.5 Procedure to account for missing data

It seems likely that some data may not be available due to voluntary withdrawal of participants, lack of completion of individual data items or general loss to follow-up. Where possible the reasons for data ‘missingness’ will be ascertained and reported. The nature and pattern of the missing-ness will be carefully considered, including whether data can be treated as missing completely at random. If judged appropriate, missing data will be imputed using the multiple imputation facilities available in the statistical analysis software.

If imputation is undertaken, the resulting imputed datasets will be analysed, together with appropriate sensitivity analyses. Any imputation methods used for scores and other derived variables will be carefully considered and justified. Reasons for ineligibility, non-compliance, withdrawal or other protocol violations will be stated and any patterns summarised. More formal

analysis, for example using logistic regression with ‘protocol violation’ as a response, may also be appropriate and aid interpretation.

6.6 Health Economic Evaluation

Prospective economic evaluation, conducted from a NHS and personal social services perspective, will be included. The economic evaluation will estimate the difference in the cost of resource inputs between the two intervention groups, enabling costs and consequences to be compared. The methods will adhere to the recommendations of the NICE Reference Case[16].

Primary research methods will be followed to estimate the costs of the treatment options, including resource inputs associated with the plaster materials and braces, supplementary interventions, adverse events and rehabilitation inputs. Broader resource utilisation associated with the ankle injury will be captured through routine health service data collection systems and participant questionnaires administered at each follow up time point.

Unit costs will be estimated from local and national sources in addition to primary research using established accounting methods. Costs will be standardised to current prices where possible. Health-related quality of life will be measured at the time of consent, and all follow time points using the EQ-5D-5L measure. Responses will be used to generate quality-adjusted life years (QALYs) using the UK time-trade-off (TTO) value set recommended by the EuroQol group [17].

Within-trial analysis using bivariate regression of costs and QALYs, with multiple imputation of missing data, will inform a probabilistic assessment of incremental treatment cost-effectiveness from a health service perspective. Missing-ness mechanisms will be explored and multiple imputation methods will be used where appropriate to avoid biases associated with complete case analysis. Costs and outcomes arising after the first year of the trial will be discounted at 3.5%. Sensitivity analyses will be undertaken to explore uncertainty on the incremental cost-effectiveness ratios and to consider issues of generalisability of the study.

More extensive economic modelling using decision-analytic methods may be considered to extend the target population, time horizon and decision context, drawing on best available information from the literature and stakeholder consultations to supplement the trial data. Parameter uncertainty in the decision-analytic model will be explored using probabilistic sensitivity analysis. Longer term costs and consequences will be discounted to present values using discount rates recommended for health technology appraisal in the UK (current discount rate: 3.5%).

7. TRIAL ORGANISATION AND OVERSIGHT

7.1 Sponsor and governance arrangements

This study will be sponsored by University of Warwick following WCTU’s SOPs.

7.2 Ethical approval

All required ethical approval(s) for the trial will be sought using the Integrated Research Application System. The trial will be conducted in accordance with all relevant regulations.

Before enrolling potential participants into the trial, each trial site must ensure that the local conduct of the trial has the agreement of the relevant NHS Trust Research & Development (R&D) department. Sites will not be permitted to enrol potential participants into the trial until written confirmation of R&D agreement is received by members of the AIR trial team within Warwick Clinical Trials Unit.

All substantial protocol amendments (e.g. changes to eligibility criteria, outcomes, analyses) will be communicated to all relevant parties (e.g. investigators, RECs, NHS Trusts, trial registries) via the TM following WCTU's SOP.

Annual reports will be submitted to the REC within 30 days of the anniversary date on which the favourable opinion was given, and annually until the trial is declared ended. REC will be notified of the end of the trial (whether at planned time or prematurely).

The CI will submit a final report to the required authorities with the results, including any publications within one year of the end of the trial.

This study design has undergone extensive peer review as part of an application process for a NIHR Career Development Fellowship that involved the following:

- Independent: A panel of at least 15 experts reviewed the study and interviewed the CI.
- Expert: Two reviewers with knowledge of the relevant discipline to consider the clinical aspects of the protocol, and/or have the expertise to assess the methodological and statistical aspects of the study also assessed this study protocol outside of the independent panel.

7.3 Trial Registration

The study will be registered with the International Standard Randomised Controlled Trial Number (ISRCTN) Register.

7.4 Notification of serious breaches to GCP and/or trial protocol

A "serious breach" is a breach which is likely to effect to a significant degree:

(a) the safety or physical or mental integrity of the subjects of the trial; or
(b) the scientific value of the trial

The sponsor and REC will be notified of any case where the above definition applies during the trial conduct phase.

7.5 Indemnity

NHS indemnity covers NHS staff, medical academic staff with honorary contracts, and those conducting the trial. NHS bodies carry this risk themselves or spread it through the Clinical Negligence Scheme for Trusts, which provides unlimited cover for this risk. The University of Warwick provides indemnity for any harm caused to participants by the design of the research protocol.

7.6 Trial timetable and milestones

	Month	Recruitment
Set-up	1-11	n/a
Recruitment	12-28	476
Follow up	29-52	n/a
Analysis	53-60	n/a

7.7 Administration

The trial co-ordination will be based at WMS/WCTU, University of Warwick.

7.8 Trial Management Group (TMG)

The Trial Management Group, consisting of the project staff and co-investigators involved in the day-to-day running of the trial, will meet regularly throughout the project. Significant issues arising from management meetings will be referred to the Trial Steering Committee or Investigators, as appropriate.

7.9 Trial Steering Committee (TSC)

The trial will be guided by a group of relevant personnel and trialists as well as at least one 'lay' representative. The TSC will have an independent Chairperson. Meetings will be held at regular intervals determined by need but not less than once a year. Routine business is conducted by email, post or teleconferencing.

The Steering Committee, in the development of this protocol and throughout the trial will take responsibility for:

- Approval of the protocol
- Major decisions such as a need to change the protocol for any reason
- Monitoring and supervising the progress of the trial
- Reviewing relevant information from other sources
- Considering recommendations from the DMC
- Informing and advising on all aspects of the trial

7.10 Data Monitoring Committee (DMC)

The DMC will consist of independent experts with relevant clinical research, and statistical experience. The DMC meeting frquency will be guided by the DMC chair, but will be suggested to be six months into the recruitment phase and regularly thereafter, as directed by the DMC chair. Confidential reports containing recruitment, protocol compliance, safety data and interim assessments of outcomes will be reviewed by the DMC. The DMC will advise the TSC as to whether there is evidence or reason why the trial should be amended or terminated.

7.11 Essential Documentation

A Trial Master File will be set up according to WMS SOP and held securely at the coordinating centre. The coordinating centre will provide Investigator Site Files to all recruiting centres involved in the trial.

7.12 Financial Support

The trial has been funded by a grant from NIHR Career Development Fellowship.

8. MONITORING, AUDIT AND INSPECTION

A Trial Monitoring Plan will be developed and agreed by the Trial Management Group (TMG) based on the trial risk assessment. We will institute a rigorous programme of quality control. The CI in conjunction with the TM will be responsible for ensuring adherence to the trial protocol at the trial sites. Quality assurance checks will be undertaken by WCTU to ensure integrity of randomisation, study entry procedures and data collection. The WCTU has a quality assurance team who will monitor this trial by conducting regular (yearly or more if deemed necessary) inspections of the Trial Master File. Furthermore the processes of consent taking, randomisation, provision of information and provision of treatment will be monitored. To achieve this the involved sites may be required to host site visits and provide information for remote monitoring.

Monitoring will initially be conducted across all sites, and subsequently conducted using a risk based approach that focuses on sites that have the highest enrolment rates, large numbers of withdrawals, or atypical (low or high) numbers of reported adverse events.

9. PATIENT AND PUBLIC INVOLVEMENT (PPI)

RG is a public representative who has volunteered to actively collaborate on the development of this protocol. RG is a UNTRAP trained member, achieving the 'Professional Development Award – User Involvement in Teaching and Research'. He will be supported by the lead applicant, Rebecca Kearney, and UNTRAP will assist in resolving any identified training needs through appropriate training events and development of good practice partnership working.

RG will be a member of the TSC, in this role he will be asked to evaluate patient facing documents such as participant information sheets and provide ongoing evaluation and commentary of key trial processes, such as obtaining informed consent. It is clear that each stage of the research will benefit from the patient and public involvement through consultation and active collaboration. Specifically this involvement will focus the research team on what is important from a patient's perspective and ensure that plain language is used in all patient resources.

10. DISSEMINATION AND PUBLICATION

The results of the trial will be reported first to trial collaborators. The main report will be drafted by the trial coordinating team, and the final version will be agreed by the Trial Steering Committee before submission for publication, on behalf of the collaboration.

The success of the trial depends on the collaboration of clinicians and researchers from across the UK. Equal credit will be given to those who have wholeheartedly collaborated in the trial.

The trial will be reported in accordance with the Consolidated Standards of Reporting Trials (CONSORT) guidelines (www.consort-statement.org).

The results of this trial will substantially inform clinical practice on the clinical effectiveness of the treatment of this injury. The results of this project will be disseminated through peer-reviewed journals, conference presentations, the National Library for Health and through local mechanisms at all participating centres.

11. APPENDICES

11.1 APPENDIX 1 – Protocol for AIR Qualitative Sub-study

Study Protocol - Qualitative Interviews Sub-Study – Ankle Injury Rehabilitation (AIR) Trial ISRCTN15537280

SUB-STUDY SUMMARY

Study Title	Qualitative Sub-Study to the AIR Trial
Study Design	Semi-structured Interviews
Study Participants	Adults with Ankle Fracture participating in the AIR Trial
Planned Size of Sample	Approximately 10 participants
Follow up duration	>16 week time point for AIR Trial
Research Question/Aim(s)	To explore the lived experiences of adults recovering from ankle fracture and make an assessment of the content validity of the questionnaires currently being used in this research area.

1 BACKGROUND

There is limited research as to the lived experiences of individuals recovering from ankle fracture and the factors most important to them in their recovery. Exploring these factors

are of high importance in clinical practice and research to ensure that outcomes being collected are significant to patients. This study aims to explore the lived experiences of adults recovering form ankle fracture and also provide some understanding of the content validity of the questionnaires used in clinical research for ankle fractures. Content validity is defined by the COSMIN Group as “the degree to which a patient reported outcome measure (PROM) is an adequate reflection of the construct to be measured” [18]. It is regarded as the most important measurement property of a PROM because it should be clear from the outset that the items being collected and measured as part of the PROM are relevant and comprehensible with respect to the population being studied [19].

12. 2 RATIONALE

The aim of this qualitative study will be to explore the lived experiences of patients recovering form ankle fracture and determine the factors most important to them in their recovery. The secondary aim of this sub-study is to determine whether the Olerud Molander Ankle Score (OMAS) questionnaire [20] is relevant and comprehensive to individuals recovering from this injury and therefore establish its content validity.

In the field of clinical research into interventions for ankle fracture, it is clear from the previous systematic review completed as part of this project that the majority of researchers are asking patients to assess their outcome, rather than relying on clinical tests and clinician assessed outcome only. However, it is apparent that the most widely used ankle specific PROM has little evidence surrounding its content validity and whether it’s assessing what is most important to individuals recovering from this injury. There is limited research on what the conceptual basis should be for a patient reported outcome measures for adults recovering from ankle fracture.

3 THEORETICAL FRAMEWORK

Evaluating the content validity of an outcome measure is of high importance and should ideally be completed prior to formulation of the outcome measure [19]. However, it has been established that this was not completed in the development of the OMAS questionnaire, which was developed without any involvement from individuals with ankle fracture. This study will aim to provide evidence for the content validity of this outcome measure from the perspective of patients recovering from this injury.

The COSMIN Group steering committee members [21] explain that establishing the content validity of an outcome measure comprises of ascertaining the relevance and comprehensiveness of the contents of the PROM to an expert panel. Relevance is concerned with whether the items of the questionnaire in its current format are relevant to the construct being tested. Comprehensiveness is concerned with the coverage of the outcome measure, so whether the questionnaire covers all aspects of construct being measured. Therefore there will be two sections to the interviews; the first will aim be to explore the lived experience and factors most important to these individuals in their injury recovery. This will be completed by conducting an exploratory interview on the lived experiences of individuals recovering from ankle fracture and the factors most important to them in their recovery. The second section will look to ask the individuals more specifically

about their thoughts of the OMAS questionnaire and the relevance of the content to them in their recovery.

13. 4 RESEARCH QUESTION/AIM(S)

Research Questions:

1. What is the lived experience and factors of most importance to a sample of adults recovering from ankle fracture?
2. What is the content validity of the Olerud Molander Ankle Score (OMAS) in a sample of adults recovering from ankle fracture?

4.1 Objectives

1. To explore the lived experiences and most important factors to adults recovering from ankle fracture through using semi structured interviews.
2. To determine whether the PROM questionnaires used in ankle fracture clinical research are relevant and comprehensive to a sample of adults with ankle fracture using semi structured interviews.

4.2 Outcome

The outcome of this project will be a qualitative exploration of the lived experiences and factors of most importance to adults recovering from ankle fracture. An assessment of the content validity of the questionnaires used in interventional trials of ankle fractures in this sample will also be made. Recommendations for outcome measurement in this population of individuals will be made, along with informing further research in this area.

14. 5 STUDY DESIGN AND METHODS OF DATA COLLECTION AND DATA ANALYIS

The objectives of this study will be achieved by using semi-structured interviews with participants taking part in the AIR study. The topic guide has been developed with supervision from senior academic supervisors with experience in qualitative research methods. The interviews will be conducted by the researcher (RM) and recorded on an encrypted, password protected audio recorder with the patient's informed consent, documented using a specific qualitative sub-study consent form.

The audio recordings will be transported back to Warwick Clinical Trials Unit on the passcode protected audio recorder and then transferred onto the University of Warwick secure servers in a secure folder accessible to only authorized trial personnel. Authorised trial personnel at Warwick CTU will be responsible for transcribing, coding and de-identifying the interviews. The data will be anonymised using trial ID and any identifiable sections of the interviews will be anonymized to protect the confidentiality of the participants involved in the study. The interviews will be recorded on a Dictaphone and then securely transferred to a password protected computer, to which the primary researcher only has access to. Following transcribing and de-identification, the researcher will then be responsible for the secure deletion of the recordings. The audio-recording will

be deleted as soon as the transcription process is completed and anonymised and saved on secure university servers. The transcriptions will be archived for 10 years as per University of Warwick CTU Standard Operating Procedures.

The data will be analysed using thematic analysis of the transcripts. The data will be coded and NVIVO software will be used to facilitate this process.

15. 6 STUDY SETTING

The interviews will be conducted in the participant's homes or at their place of work in a quiet location which allows for the interview to take place with minimal distractions. Research into qualitative interview methodology in health research has shown that it is favourable to perform this type of interview in the participant's own environment [19]. The interviews will be conducted at an appropriate time most convenient for the participant.

The interviews will be approximately one hour in length and will be completed using the AIR Qualitative sub-study Interview Guide (Version 1.0 and 22/08/2018) in order to standardise the prompts given to each participant and guide the conversation in a semi-structured way.

7 SAMPLE AND RECRUITMENT

7.1 Eligibility Criteria

7.1.1 Inclusion criteria

As per AIR main study Eligibility Criteria as these individuals will already be participating in the AIR Study.

And participants who have selected "Yes" to item 6 of the AIR Consent form, relating to being contacted about further research in this area.

7.1.2 Exclusion criteria

As per AIR main study Eligibility Criteria as these individuals will already be participating in the AIR Study.

And participants who have selected "No" to item 6 of the AIR Consent form, relating to being contacted about further research in this area. Participants who have not yet completed their 16 week follow up questionnaire will be excluded so that any added effects of the interviews does not contaminate the primary outcome of the main trial.

7.2 Sampling

7.2.1 Size of sample

The approximate sample size for this study is 10. The decision on when to stop or continue with further interviews will be when data saturation has been reached and no further topics emerge from the interviews. The time frame for this interview will ensure that the main study is not contaminated by the interview, therefore all participants taking part in

the qualitative study will have completed their primary outcome time point questionnaire (16 weeks).

7.2.2 Sampling technique

This project will utilise purposive sampling to gather data from a range of participants in the AIR trial, taking into account variables which may affect responses. These three variables are age, gender and fracture management (operatively managed or non-operatively managed). The sample will have a minimum number individuals from each of the groups to account for these variables. There will be a minimum of two participants in each age group interviewed. Each age group will be made up of at least one male and one female. Furthermore, in each age group there will be a mixture of patients fracture management, so a mixture (at least one of each) operatively managed conservatively managed patients. This would be the ideal situation, however the research team acknowledge that there may be logistical issues with this sampling technique. Response to interview invites is not something that can be controlled, therefore we will adapt this accordingly as necessary.

7.3 Recruitment

7.3.1 Sample identification

Participants in the AIR Trial who have selected that they are willing to be contacted about future research on their consent form will be screened using a report on the AIR Online Application, as explained in section 7.1.

The participant's demographics will also be reviewed during this process, to ensure the purposive sampling is followed with respect to their age, gender and fracture management.

This screening will be completed by the researcher using the AIR Online Application which is a secure web based database accessible only to authorised trial personnel, as detailed in the AIR Data Management Plan.

Participants who fulfil the eligibility criteria and the criteria for purposive sampling will be sent a Participant Information Sheet (V1.0 22/08/2018) for the Qualitative Interview Sub-Study which will outline the study procedures. An accompanying cover letter will explain to the participant that a researcher will be in touch with them via telephone to discuss this in further detail in 1 weeks' time.

During this phone call, the researcher will discuss the study in more detail with the participant, giving time for questions to be asked and appropriately answered. If the participant is happy to take part, then an interview will be scheduled over the telephone. The participants will be given a choice to complete the interview in their own home, their place of work or another mutually agreed appropriate location.

7.2.2 Consent

Written informed consent will be gained from participants prior to under taking any research activities related to this embedded study.

The consent form will then be completed in person prior to the start of the interview at the scheduled appointment. During this time, there will also be another opportunity to discuss the study in further details or have any other questions answered. The researcher will make the participant aware of the intention to record the interview prior to beginning and also make the participant aware of their rights, which is that they are able to withdraw consent at any time and also have the right to have the recording deleted following the interview should they wish this to happen.

As per the eligibility criteria for the AIR trial, all participants included in the main study will have capacity. However, there is a small chance that the patient has lost capacity since entering the AIR study, so the researcher will ensure that capacity is assessed by ensuring that the participant understands the purpose and the nature of the research, understands the processes involved and be able to retain information on the study prior to gaining consent.

Prior to beginning any study related activities, the participant will provide written informed consent, which will be countersigned by the researcher obtaining the consent as per HRA guidelines.

16. 8 ETHICAL AND REGULATORY CONSIDERATIONS

There is no identified benefits to the participant taking part in this study, other than that their contribution will be helping to contribute to the evidence base in the area. There is no expected risk of harm to participants taking part in this project. The data collection method will uphold the dignity of the participants as all transcribing will be completely anonymised and any quotations will not be attributed to the individual. The participant will be free to withdraw their consent at any time up until their data is anonymised without having to give a reason. They will be made aware that they are able to request that the recording of the interview be deleted up to 72 hours following their interview, should they wish to for any reason. Once the interview has been transcribed and anonymised, the recording will be deleted and after this the participant will not be able to withdraw their data as it has already been anonymised.

16.1 8.1 Assessment and management of risk

This is a low risk project and there is no expected risk of harm to participants. There is a potential risk to the researcher completing research activities in a lone working environment in participants own homes. The Lone Working Guidelines developed by Warwick CTU Quality Assurance Team will be followed, to maintain researcher safety at all times. An Individual Worker Risk Assessment for Home Visits will be completed prior to undertaking the interviews to ensure that risks are adequately managed during this research project.

8.2 Research Ethics Committee (REC) review & reports

Before the start of the study, approval will be sought from the Research Ethics Committee (REC) and the Health Research Authority (HRA) via an amendment and appendix to the AIR

Protocol. The qualitative study protocol, informed consent forms and patient information sheet will all be submitted for review and approval prior to starting study activities.

Correspondence with the REC will be retained by the study team. There will be annual progress reports submitted as part of the AIR Trial. The study team will notify the REC at the end of the study. If the study is ended prematurely, the study team will notify the REC with reasons for the premature termination. Within a year following the end of the study, the study team will submit a final report with the results, including any publications/abstracts.

8.3 Patient & Public Involvement

As per the AIR Protocol, this appendix protocol for qualitative research has been reviewed by the AIR study PPI Representatives (KK and RG), have reviewed and commented on the acceptability, design and undertaking of the research. They will also take an active role in the analysis of results and dissemination of the findings through review and approval of outputs of the research.

8.4 Regulatory Compliance

This embedded qualitative project will not involve participating trial sites or affect the NHS R&D Permissions to complete the research activities.

8.5 Protocol compliance

Any deviations from the approved protocol which occur will be documented on a protocol deviation form and reported to the Chief Investigator. Serious non-compliances and breaches of the protocol will be reported to the University of Warwick Sponsorship Team (Sponsor) and WCTU Quality Assurance Team and appropriate actions will be determined and reported to the authorities if required.

16.1.1

8.6 Data protection and patient confidentiality

All investigators will comply with the requirements of the Data Protection Act 2018 and any other relevant legislation with regards to the collection, storage, processing and disclosure of personal information and will uphold the Act's core principles.

The data collected here will be recorded on a passcode protected audio recorder. The interviews will then be transferred to the University of Warwick secure servers, transcribed, coded and anonymised by trial ID number. The audio-recordings will then be securely deleted following transcription and anonymisation. The anonymised transcribed data will be held in a secure password protected computer, accessible only to authorised trial personnel. This data will be archived for 10 years following study completion, as per WCTU Standard Operating Procedures.

8.7 Indemnity

This research project will be covered under the Insurance held for the AIR Trial, provided with the initial application of this project.

8.8 Amendments

Any amendments to this protocol will be submitted to the REC and HRA via the standard process.

8.9 Access to the final study dataset

As outlined in the main protocol for AIR.

16.1.2 9 DISSEMINIATION POLICY

16.1.3 9.1 Dissemination policy

Upon completion of the study, the data will be analysed and tabulated and a Final Study Report prepared. The full study report will be accessible on the AIR Main Webpage on the University of Warwick Clinical Trials Unit Webpage. The investigators will publish the study in journals and present the research at academic meetings but none of the data will be identifiable or attributable to individual.

16.2 APPENDIX 2 – Protocol for AIR Sub-study 2

Sub-study Protocol – A Study of the Validity, Reliability, Responsiveness and Interpretability of the Olerud Molander Ankle Score.

SUB-STUDY SUMMARY

Study Title	A study of the validity, reliability, responsiveness and interpretability study of the Olerud Molander Ankle Score.
Study Design	Study of measurement properties
Study Participants	Participants included in the AIR trial – secondary analysis of pre-existing data
Planned Size of Sample	Approximately 300-400
Follow up duration	N/A
Research Question/Aim(s)	What is the validity, reliability, responsiveness and interpretability of the Olerud Molander Ankle Score in adults with ankle fracture?

Background

A systematic review of measurement properties of outcome measures for ankle fracture highlighted the lack of evidence for the validity, reliability and responsiveness of the outcome measures used for this patient population {Ng, 2018 #6028}. This project will contribute to the evidence base regarding the validity, reliability, responsiveness and interpretability of the Olerud Molander Ankle Score (OMAS) in the population of adults with ankle fracture.

The terminology and measurement property definitions used in this project will be in accordance with the COSMIN Group published work in this field {Mokkink, 2010 #6891}.

Participants

This project will utilise secondary analyses of pre-existing data collected in the AIR Trial and will not involve further contact with participants outside of follow up data currently collected for the AIR trial.

The data for all individuals who have consented to randomisation and follow up as part of the AIR trial will be eligible for inclusion in this study.

Sample Size

The sample size for this project will be approximately 300-400, depending upon the number of participants for which complete data sets exist for follow up time points necessary for analysis. Recommend sample sizes for studies on measurement properties are a minimum of 50 but ideally over 100 {De Vet, 2011 #6877} therefore a larger number has been chosen here to fulfil this criteria.

Statistical Analysis

Further details of analyses performed as part of this sub-study will be included a more detailed Statistical Analysis Plan (SAP) for this sub-study, which will be reviewed and approved by the AIR Trial Management Group (TMG) prior to commencing data analysis.

The data required for this sub-study will be accessed with the permission of the TMG and this approval will be recorded in the TMG meeting minutes. The trial statistician or nominated delegate will retrieve the data from the AIR online application. This will be transferred to an Excel Spreadsheet and deposited to a file on the AIR Shared folders (M:Drive) on secure University of Warwick servers' access to which is only permitted to authorised trial personnel. All data will be anonymised by trial ID and there will be no identifying patient information on any of the data. At the end of the project, the data will be archived along with the AIR shared folders outlined in the main protocol for the AIR trial.

Data required for this study will be as follows:

1. Patient demographics – Age (y), Age group (49 years and under or 50 years and over), gender, mechanism of injury, side of fracture, weber Classification, number of malleoli involved in fracture, operative or conservative management of fracture.
2. OMAS scores at baseline pre-injury, post-injury, 6 weeks, 10 weeks and 16 weeks.
3. DRI, EQ-5D-5L and M-OXFQ scores for baseline pre-injury, post-injury, 6 weeks, 10 weeks and 16 weeks.
4. The Global Impression of Change Score at the 16 week time point.

There will be no analysis and therefore requirement of data regarding the treatment allocation arm involved in this sub-study. Exact variables required for this sub-study will be outlined in the sub-study SAP.

Objectives:

1. To assess the structural validity of the OMAS in a population of adults with ankle fracture participating in the AIR trial.
2. To determine the internal consistency of the OMAS in a population of adults with ankle fracture participating in the AIR trial.

3. To determine the construct validity/convergent validity of the OMAS in relation to the DRI, the M-OXFQ and the EQ-5D-5L in a population of adults with ankle fracture participating in the AIR trial.
4. To ascertain the construct validity (discriminant validity) the OMAS between different fracture types and fracture managements in a population of adults with ankle fracture participating in the AIR trial.
5. To assess the responsiveness of the OMAS in a population of adults with ankle fracture participating in the AIR trial.
6. To assess the interpretability of the OMAS in a population of adults with ankle fracture participating in the AIR trial.

Structural Validity

Structural validity of the OMAS will be determined using Exploratory Factor Analysis (EFA). Exploratory factor analysis will be performed to investigate the unidimensionality of the OMAS i.e. whether the PROM measures a single measureable construct {De Vet, 2011 #6877}.

Reliability - Internal Consistency

Internal consistency is the degree of interrelatedness amongst the items in an outcome measure or the extent to which the items on a questionnaire all measure the same construct {Mokkink, 2010 #6891}

Cronbach's Alpha will be used to determine the internal consistency of items in the OMAS questionnaire.

Construct Validity

Construct Validity is defined as the degree to which the scores of a PROM are consistent with the hypotheses (in regard to internal relationships, relationships to scores of other instruments, or differences between relevant subgroups) based on the assumption that the PROM validly measures the construct to be measured {De Vet, 2011 #6877}.

For this analysis, OMAS scores will be correlated with other PROMS which are collected as part of the AIR Trial. The PROMS used will be the Manchester Oxford Foot Questionnaire (M-OXFQ), The Disability Rating Index (DRI) and the EQ-5D-5L. The correlations will be performed following formulation of a set of hypotheses regarding the scale of correlation expected with each PROM. These hypotheses will be outlined in the SAP for this sub-study.

Assuming the data are normally distributed, Pearson's Correlation Coefficient will be used to correlate these scores.

Three further analyses will be involved in understanding the discriminant validity of the OMAS by assessing the average scores in clinically different groups:

1. Difference in OMAS scores between patients with Weber A, B and C fractures
2. Difference in OMAS scores between patients with Uni, Bi and Tri malleolar fractures
3. Difference in OMAS between operatively and non-operatively managed patients

Responsiveness

Responsiveness is defined as the ability of a PROM to detect change over time when one occurs {De Vet, 2011 #6877}

The responsiveness of the OMAS and MOXFQ will be ascertained by using the Global Impression of Change (GIC) Score. This question is asked at 16 week time point, asking the individual to indicate how their ankle is compared to the last follow up time point (10 week follow up time point). Individuals will be grouped into 8 different groups depending upon their answer to the GIC score question:

1. Very Much Improved
2. Much Improved
3. Improved Minimally
4. No Change
5. Minimally worse
6. Much Worse
7. Very Much worse

For each of these groups, the mean change in OMAS scores between the 10 and 16 week time points will be calculated and displayed on a boxplot graph to demonstrate the changes in OMAS for each of the eight groups of GIC score responses.

Interpretability

Interpretability is defined as the degree wo which one can assign qualitative meaning – that is, clinical or commonly understood connotations – to an instrument's quantitative scores or change in scores' {Mokkink, 2010 #6891}.

To assess interpretability, means and standard deviations of OMAS Scores at follow up time points will be ascertained. Then an evaluation of floor and ceiling effects will be completed at each time point on the overall OMAS and on the individual items of the OMAS. Floor effects will be defined as a proportion of ≥15% of participants achieving the lowest score (0) on the OMAS questionnaire and ceiling effects will be defined as a proportion of ≥15% of participants achieving the highest score (100) on the OMAS questionnaire, which has been recommended and followed by other researchers {Terwee, 2007 #3060}, {Wamper, 2010 #6885}. An assessment of the Minimally Important Change (MIC) will also be made by using anchor-based methods. The anchor will be the Global Impression of change score.

Dissemination of Results

Upon completion of the study, the data will be analysed and tabulated and a Final Study Report prepared. The full study report will be accessible on the AIR Main Webpage on the University of Warwick Clinical Trials Unit Webpage. The investigators will publish the study in journals and present the research at academic meetings but none of the data will be identifiable or attributable to individual participants.

Appendix 3 – Summary of changes to protocol

**Summary of Changes to Protocol V6.0 01/04/2019**		
**Page**	**Section**	**Summary of change**
45-47	Appendix 2	Inclusion of sub-study to assess validity, reliability, responsiveness and interpretability of OMAS
**Summary of Changes to Protocol V4.0 22/08/2018**		
**Page**	**Section**	**Summary of change**
28	5.1	Inclusion of £5 patient voucher in the follow up questionnaires
36-43	Appendix 1	Inclusion of Qualitative sub-study to complete semi-structured interviews with a sample of AIR Participants

**Summary of Changes to Protocol v3.0 11/05/2018**		
**Page**	**Section**	**Summary of change**
2	N/A	Change of CI – Return of CI from Maternity Leave
2	N/A	Change of Address and Telephone number of Trial Manager and team to Clinical Sciences Building
2	N/A	Change of Trial co-ordinator title to Trial Manager
20	Section 2.2.2	Removal of “Following Surgical Management” from exclusion

Summary of Changes to Protocol v3.0 11/05/2018		
**Page**	**Section**	**Summary of change**
		criteria point 5.
36	11	Addition of section 11 to record summaries of changes to protocol

Summary of Changes to Protocol v2.0 09/10/2017		
**Page**	**Section**	**Previous wording**
25/26	3.1	Change in the complications being recorded to include collection of X-Rays at baseline and the last one taken up to the 16 week time point (if taken)
2	N/A	Change in sponsor contact email address

17. REFERENCES

1. Court-Brown, C.M. and B. Caesar, *Epidemiology of adult fractures: A review.* Injury, 2006. **37**(8): p. 691-7.
2. Kannus, P., et al., *Increasing number and incidence of low-trauma ankle fractures in elderly people: Finnish statistics during 1970-2000 and projections for the future.* Bone, 2002. **31**(3): p. 430-3.
3. Murray, A.M., et al., *Cost description of inpatient treatment for ankle fracture.* Injury, 2011. **42**(11): p. 1226-9.
4. McPhail, S.M., et al., *Life impact of ankle fractures: qualitative analysis of patient and clinician experiences.* BMC Musculoskelet Disord, 2012. **13**: p. 224.
5. Donken, C.C., et al., *Surgical versus conservative interventions for treating ankle fractures in adults.* Cochrane Database Syst Rev, 2012(8): p. CD008470.
6. Hedstrom, M., T. Ahl, and N. Dalen, *Early postoperative ankle exercise. A study of postoperative lateral malleolar fractures.* Clin Orthop Relat Res, 1994(300): p. 193-6.
7. Willett, K.M., et al., *Orthopaedic trauma research priority-setting exercise and development of a research network.* Injury, 2010. **41**(7): p. 763-7.
8. Lin, C.W., et al., *Rehabilitation for ankle fractures in adults.* Cochrane Database Syst Rev, 2012. **11**: p. CD005595.
9. Kearney, R.S., et al., *A protocol for a feasibility randomised controlled trial to assess the difference between functional bracing and plaster cast for the treatment of ankle fractures.* Pilot Feasibility Stud, 2017. **3**: p. 11.

10. Olerud, C. and H. Molander, *A scoring scale for symptom evaluation after ankle fracture.* Arch Orthop Trauma Surg, 1984. **103**(3): p. 190-4.
11. Herdman, M., et al., *Development and preliminary testing of the new five-level version of EQ-5D (EQ-5D-5L).* Qual Life Res, 2011. **20**(10): p. 1727-36.
12. Salen, B.A., et al., *The Disability Rating Index: an instrument for the assessment of disability in clinical settings.* J Clin Epidemiol, 1994. **47**(12): p. 1423-35.
13. Berntsen, G.K., et al., *Forearm bone mineral density by age in 7,620 men and women: the Tromso study, a population-based study.* Am J Epidemiol, 2001. **153**(5): p. 465-73.
14. Willett, K., et al., *Close Contact Casting vs Surgery for Initial Treatment of Unstable Ankle Fractures in Older Adults: A Randomized Clinical Trial.* JAMA, 2016. **316**(14): p. 1455-1463.
15. Lamb, S.E., et al., *Mechanical supports for acute, severe ankle sprain: a pragmatic, multicentre, randomised controlled trial.* Lancet, 2009. **373**(9663): p. 575-81.
16. NICE, *Guide to the methods of technology appraisal 2013.* London: National Institute of Health and Care Excellence., 2013.
17. Oppe, M., et al., *EuroQol Protocols for Time Trade-Off Valuation of Health Outcomes.* Pharmacoeconomics, 2016. **34**(10): p. 993-1004.
18. Mokkink, L.B., et al. *COSMIN methodology for systematic reviews of patient reported outcome measures (PROMs) - user manual*. 2018 03/07/2018]; Available from: https://cosmin.nl/wp-content/uploads/COSMIN_manual_syst-review-PROMs_V1.0.pdf.
19. Sim, J. and C. Wright, *Research in Health Care*. 2000, Cheltenham: Stanley Thornes.
20. Olerud, C. and H. Molander, *A Scoring Scale for Symptom Evaluation After Ankle Fracture*. Archives of Orthopaedic and Traumatic Surgery, 1984. **103**.
21. De Vet, H.C.W., et al., *Measurement in Medicine*. 2011, New York, USA: Cambridge University Press.
