# Supplementary material for: Use of cast immobilisation versus removable brace in adults with an ankle fracture: multicentre randomised controlled trial
Source: BMJ. 2021 Jul 6;374:n1506. doi: 10.1136/bmj.n1506 (PMC8256800; doi:10.1136/bmj.n1506)
Supplement: Supplementary file 2 — Supplementary information: file 2 [file kear065381.ww2.pdf]

<<Local Trust Logo>>

Functional Brace Exercise Sheet

You have been randomised to receive the functional brace as part of the Ankle Injury Rehabilitation Study. As part of this treatment, we would like to you remove your boot to complete some exercises. The following exercises are to be completed little and often, as comfort allows. We recommend 10 times each, 3 times a day.

1.

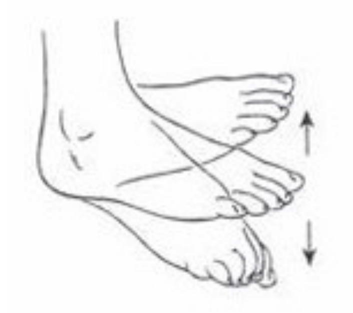

- Lying on your back or sitting.
- Bend and straighten your ankle as far as you can.

2.

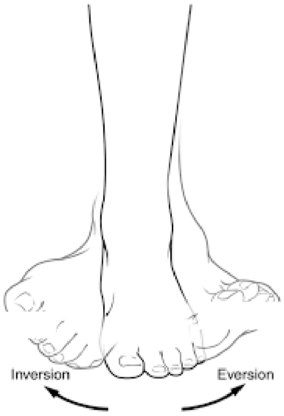

- Sitting with your foot on the floor.
- Alternately raise the inner border of your foot (big toe) and then the outer border (little toe) as much as you can.

This research was funded by the NIHR Career Development Fellowship (Ref: CDF-2016-09-009)
