# Supplementary material for: Use of cast immobilisation versus removable brace in adults with an ankle fracture: multicentre randomised controlled trial
Source: BMJ. 2021 Jul 6;374:n1506. doi: 10.1136/bmj.n1506 (PMC8256800; doi:10.1136/bmj.n1506)
Supplement: Supplementary file 3 — Supplementary information: file 3 [file kear065381.ww3.pdf]

Supplement Three

Additional sensitivity, subgroup and secondary analysis

OMAS Sensitivity Analyses

Fixed effect model

As previously described in the main analyses, some of the sensitivity and secondary analyses also failed to converge using the random effects model with site as the random intercept. This is due to a degree of collinearity been the site of randomisation and fracture management: four sites only randomised operatively managed patients (two sites) or non-operatively managed patients (two sites).

The main random effect model was tested against the fixed effect model to find out if it had improved the fit of the data, and the estimate of the treatment effect. It was found that the addition of the random effect model was did not improve the model fit. That is, the nested models were not significantly different (likelihood ratio test, p=0.3436). Hence, for parsimonious reasons, only the fixed effect models are reported in the main results.

Table1 shows the results of the fixed effect model.

Table 1: Adjusted fixed effect model, the model has been adjusted for patient gender, age group and fracture management

Model variable (N=502)		Coefficient	95% CI	p-value
Intervention Group	Cast	0	-	0.357
	Removable Brace	1.8	(-2.0 to 5.6)	
Gender	Female	0	-	p <0.001
	Male	7.7	(3.7 to 11.5)	
Fracture Managed	Operatively	0	-	p <0.001
	Non - operatively	11.9	(8.0 to 15.7)	
Age group	49 & under	0	-	0.005
	Over 49	-5.5	(-9.4 to -1.7)	

Baseline adjustment

On inspection, baseline (post injury) OMAS scores appeared balanced between allocation groups (main paper, Table 1) but exhibited some floor effects, with n=71 (11% of valid responses) scoring the minimum possible. Thus, baseline scores did not meet the criteria to be added as an additional model covariate and was omitted from the final analysis. However, we have investigated the effects of including baseline as a sensitivity analysis in Table 2 below.

Table 2: Adjusted fixed effect model, the model has been adjusted for baseline OMAS score, patient gender, age group and fracture management

Model variable (N=502)		Coefficient	95% CI	p-value
Intervention Group	Plaster Cast	0	-	0·424
	Functional Brace	1.5	(-2.2 to 5.3)	
Gender	Female	0	-	p <0·001
	Male	7·0	(3.2 to 10.9)	
Fracture Managed	Operatively	0	-	p <0·001
	Non - operatively	13.3	(9.5 to 17.1)	
Age group	49 & under	0	-	p<0.001
	Over 49	-7.2	(-11.0 to -3.3)	
OMAS baseline score		0.3	(0.2 to 0.4)	p<0.001

Multiple imputation

As loss to follow up was higher than the expected 20% at the primary analysis point, multiple imputation using chain equations was used to investigate the robustness of the results. Missingness of the primary outcome was not evenly distributed between allocation groups, with more data missing in the cast group (completeness of cast 73%; removable brace 78%).

Twenty five imputed data sets were created for each model and the coefficients were pooled using Rubin's rules used to combine estimates. Missing OMAS scores were imputed using predictive mean matching (pmm) method. Variables were chosen from the baseline and randomisation set based upon their association with missingness at 16 week follow up. The following predictor variables were chosen:

- Baseline variables: fractures on patients medial and posterior malleolus; concurrent injuries on the same leg; participants weight and if participants smoke.
- Stratification variables: age group, gender and fracture management.

The sensitivity analyses on the imputed data set gave similar results to those in the primary analysis. The conclusion is that missingness does not impact on the interpretation of the primary analysis.

Table 3: MI pooled estimated for the adjusted RE model

Variable		Coefficients	95% C.I	p-value
Intervention	Cast	0	-	0.254
	Removable Brace	2.2	(-1.6 to 6.1)	
Gender	Female	0	-	p <0.001
	Male	7.5	(3.7 to 11.3)	
Fracture Managed	Operatively	0	-	p <0.001
	Non – operatively	12.4	(8.4 to 16.3)	
Age Group	49 and Under	0	-	0.003
	Over 49	-5.7	(-9.4 to -1.9)	

Intervention adherence

Table4 shows the protocol deviations and violations related to adherence that occurred during the study. A cross over was considered a deviation if it occurred within three weeks of randomisation. One participant crossed groups (brace to cast) and was considered a protocol violation as the participant was not correctly consented to take part in the study.

Table 4: Protocol deviations related to adherence

Adherence outcomes	Cast (n)	Brace (n)	Total (n)
Total adherence deviations and violations	23	7	30
Crossed over: patient decision	20	4	24
Crossed over: clinician decision	1	2	3
No intervention worn	2	1	3

In this pragmatic trial, any other rehabilitation input beyond rigid immobilisation in a cast vs. early active movement in a removable brace was at the discretion of the treating member of the clinical team. This included choice of weight bearing status for the participant, exact duration of immobilisation period (beyond the minimum of three weeks) and decision to onward refer participants to physiotherapy services.

In the cast group 78 participants (n=334) reported remaining in cast at six weeks, reducing to 47 at 10 weeks and eight at 16 weeks. In the removable brace group 151 participants (n=335) reported remaining in their removable brace at six weeks, reducing to 50 at 10 weeks and 11 at 16 weeks.

Initial advised weightbearing status similar across trial arms; with weight bearing allowed in 110 participants in the cast group and 109 in the removable brace group. There was also a similar number of onward referrals to physiotherapy in both groups, with 182 in the cast group and 166 in the removable brace group.

Per protocol analysis

A per protocol analysis was conducted, removing those who did not wear their intervention for three weeks. However, the random effects model did not converge, due to collinearity between the fracture management and site of recruitment variables. A fixed effect model for the per protocol is shown in Table5. The per protocol model remained similar to the primary analysis.

Table 5: Adjusted model with fixed effects only for the per protocol analysis

Variable (n = 499)		Coefficients	95% C.I	p-value
Intervention	Cast	0	-	0.494
	Removable Brace	1.3	(-2.5 to 5.2)	
Gender	Female	0	-	p <0.001
	Male	7.4	(3.5 to 11.3)	
Fracture Managed	Operatively	0	-	p <0.001
	Non – operatively	12.1	(8.2 to 15.9)	
Age Group	49 and Under	0	-	0.002
	Over 49	-6.1	(-9.9 to -2.2)	

OMAS Subgroup Analyses

Sub-group analyses were undertaken to assess whether there is evidence that the intervention effect for operatively managed participants and non-operatively managed participants; as well as for the under and over 50 year olds. Note that the study is not powered to detect subgroup effects, so p-values have been omitted from the model tables in this section. However, the 95% confidence intervals have been calculated.

Fracture management

For the fracture management subgroup, a random effects model did not converge, so a fixed effect model omitting the site of randomisation was constructed. An ANOVA comparing the models with and without the interaction variable (see above for details). Table6 shows that the two models are not significantly different (F=1.1038, p=0.294).

Table 6: Subgroup analysis: Fracture management model

Variable (N=502)		Coefficient	95% C.I
Allocation group	Cast	0	-
	Removable Brace	3.7	(-1.5 to 8.9)
Fracture Management	Operatively	0	-
	Non – operatively	14.0	(8.5 to 19.5)
Interaction: group*management		-4.1	(-11.8 to 3.6)
Gender	Female	0	(3.8 to 11.7)
	Male	7.7	
Age Group	49 and Under	0	(-9.3 to -1.6)
	Over 49	-5.4	

Age group

Again, for the age group subgroup, a random effects model did not converge, so a fixed effect model omitting the site of randomisation was constructed. An ANOVA comparing the models with and without the interaction variable (Table 7) showed that the two models are not significantly different (F=0·132, p=0·717).

Table 7: Subgroup analysis: Age group model

Variable (N=502)		Coefficient	95% C.I
Allocation group	Cast	0	-
	Removable Brace	1.1	(-4.4 to 6.5)
Age Group	49 and Under	0	-
	Over 49	-6.3	(-11.8 to -0.7)
Interaction: group*age		1.4	(-6.2 to 9.1)
Gender	Female	0	(3.7 to 11.6)
	Male	7.7	
Fracture Management	Operatively	0	(8.0 to 15.7)
	Non – operatively	11.8	

OMAS Secondary Analyses

Likelihood ratio tests were used to determine if the random effect models and the fixed effect model with the 'site' variable were an improvement of the standard adjusted linear model. Again, the addition of the random effects did not improve fit, hence only the fixed effect models are shown.

OMAS Area Under the Curve (AUC)

The AUC has been calculated for all participants who have at least baseline (post injury) and 16 week follow up data; using the number of weeks from randomisation as the abscissa. The AUC was calculated using linear

interpolation for participants missing either the week 6 or 10 follow up and scaled such that the AUC also lies on a 0 – 100 point scale. Table 8 shows the descriptive statistics for the AUC.

Table 8: AUC descriptive statistics and unadjusted difference.

	Cast	Brace	Difference (95% CI)	p-value
N	194	211	-	-
AUC (Mean, SD)	42.2 (16·3)	44.1 (17.0)	1.8 (-1.4,5.1)	0.266

Table 9: ***Adjusted AUC model***

Variable		Coefficients	95% CI	p-value
Intervention	Cast	0	-	0.299
	Removable Brace	1.6	(-1.5 to 4.1)	
Gender	Female	0	-	p <0.001
	Male	5.7	(2.5 to 8.9)	
Fracture Managed	Operatively	0	-	p <0.001
	Non – operatively	9.9	(6.8 to 13.0)	
Age Group	49 and Under	0	-	0.723
	Over 49	-0.5	(-3.8 to 2.6)	

EQ-5D-5L

Individual EQ-5D-5L domains were presented in numbers and percentages and analysed with Fisher's exact test.

Table 10: EQ5D Domains, values are presented in numbers and percentages·

EQ5D Domain	Level	Pre Injury		Baseline		Six Week		Ten Week		Sixteen Week	
		Cast	Brace	Cast	Brace	Cast	Brace	Cast	Brace	Cast	Brace
	Level 1	255 (76·6%)	253 (75·5%)	155 (46·7%)	158 (47·4%)	110 (45·6%)	121 (46·9%)	125 (54·3%)	142 (59·4%)	142 (58·7%)	173 (66·8%)
	Level 2	44 (13·2%)	51 (15·2%)	100 (30·1%)	100 (30·0%)	77 (32·0%)	92 (35·7%)	69 (30·0%)	64 (26·8%)	64 (26·4%)	54 (20·8%)
	Level 3	23 (6·9%)	17 (5·1%)	54 (16·3%)	47 (14·1%)	41 (17·0%)	36 (14·0%)	29 (12·6%)	27 (11·3%)	25 (10·3%)	25 (9·7%)
**Anxiety**	Level 4	7 (2·1%)	10 (3·0%)	15 (4·5%)	11 (3·3%)	10 (4·1%)	5 (1·9%)	4 (1·7%)	5 (2·1%)	7 (2·9%)	3 (1·2%)
	Level 5	3 (0·9%)	4 (1·2%)	8 (2·4%)	16 (4·8%)	3 (1·2%)	4 (1·6%)	2 (0·9%)	1 (0·4%)	3 (1·2%)	4 (1·5%)
	Missing	1 (0·3%)	(0·0%)	(0·0%)	1 (0·3%)	(0·0%)	(0·0%)	1 (0·4%)	(0·0%)	1 (0·4%)	(0·0%)
	p-value*	0·720		0·709		0·443		0·493		0·823	
	Level 1	296 (88·9%)	289 (86·3%)	6 (1·8%)	5 (1·5%)	23 (9·5%)	18 (7·0%)	40 (17·4%)	43 (18·0%)	80 (33·1%)	101 (39·0%)
	Level 2	17 (5·1%)	16 (4·8%)	16 (4·8%)	17 (5·1%)	56 (23·2%)	82 (31·8%)	83 (36·1%)	104 (43·5%)	98 (40·5%)	95 (36·7%)
	Level 3	11 (3·3%)	15 (4·5%)	73 (22·0%)	64 (19·2%)	95 (39·4%)	91 (35·3%)	83 (36·1%)	71 (29·7%)	46 (19·0%)	55 (21·2%)
**Mobility**	Level 4	7 (2·1%)	11 (3·3%)	113 (34·0%)	102 (30·6%)	42 (17·4%)	39 (15·1%)	21 (9·1%)	18 (7·5%)	16 (6·6%)	8 (3·1%)
	Level 5	1 (0·3%)	3 (0·9%)	124 (37·3%)	144 (43·2%)	25 (10·4%)	28 (10·9%)	2 (0·9%)	3 (1·3%)	1 (0·4%)	(0·0%)
	Missing	1 (0·3%)	1 (0·3%)	(0·0%)	1 (0·3%)	(0·0%)	(0·0%)	1 (0·4%)	(0·0%)	1 (0·4%)	(0·0%)
	p-value*	0·642		0·591		0·267		0·429		0·156	
	Level 1	280 (84·1%)	275 (82·1%)	26 (7·8%)	18 (5·4%)	17 (7·1%)	19 (7·4%)	22 (9·6%)	21 (8·8%)	44 (18·2%)	49 (18·9%)
	Level 2	29 (8·7%)	27 (8·1%)	78 (23·5%)	72 (21·6%)	118 (49·0%)	134 (51·9%)	110 (47·8%)	133 (55·6%)	122 (50·4%)	136 (52·5%)
	Level 3	13 (3·9%)	19 (5·7%)	152 (45·8%)	162 (48·6%)	88 (36·5%)	89 (34·5%)	86 (37·4%)	71 (29·7%)	64 (26·4%)	60 (23·2%)
**Pain**	Level 4	9 (2·7%)	9 (2·7%)	56 (16·9%)	63 (18·9%)	15 (6·2%)	14 (5·4%)	11 (4·8%)	12 (5·0%)	10 (4·1%)	13 (5·0%)
	Level 5	1 (0·3%)	3 (0·9%)	20 (6·0%)	18 (5·4%)	3 (1·2%)	2 (0·8%)	(0·0%)	2 (0·8%)	1 (0·4%)	1 (0·4%)
	Missing	1 (0·3%)	2 (0·6%)	(0·0%)	(0·0%)	(0·0%)	(0·0%)	1 (0·4%)	(0·0%)	1 (0·4%)	(0·0%)
	p-value*	0·704		0·65		0·941		0·255		0·911	
	Level 1	314 (94·3%)	313 (93·4%)	54 (16·3%)	52 (15·6%)	115 (47·7%)	143 (55·4%)	170 (73·9%)	181 (75·7%)	210 (86·8%)	231 (89·2%)
	Level 2	6 (1·8%)	9 (2·7%)	89 (26·8%)	75 (22·5%)	69 (28·6%)	71 (27·5%)	42 (18·3%)	45 (18·8%)	22 (9·1%)	21 (8·1%)
	Level 3	7 (2·1%)	9 (2·7%)	115 (34·6%)	137 (41·1%)	42 (17·4%)	35 (13·6%)	14 (6·1%)	12 (5·0%)	7 (2·9%)	6 (2·3%)
**Self Care**	Level 4	4 (1·2%)	4 (1·2%)	56 (16·9%)	54 (16·2%)	15 (6·2%)	9 (3·5%)	2 (0·9%)	1 (0·4%)	1 (0·4%)	1 (0·4%)
	Level 5	1 (0·3%)	(0·0%)	18 (5·4%)	14 (4·2%)	(0·0%)	(0·0%)	1 (0·4%)	(0·0%)	1 (0·4%)	(0·0%)
	Missing	1 (0·3%)	(0·0%)	(0·0%)	1 (0·3%)	(0·0%)	(0·0%)	1 (0·4%)	(0·0%)	1 (0·4%)	(0·0%)
	p-value*	0·838		0·458		0·194		0·876		0·895	
	Level 1	305 (91·6%)	297 (88·7%)	7 (2·1%)	5 (1·5%)	26 (10·8%)	21 (8·1%)	46 (20·0%)	50 (20·9%)	79 (32·6%)	93 (35·9%)
	Level 2	12 (3·6%)	14 (4·2%)	28 (8·4%)	21 (6·3%)	49 (20·3%)	67 (26·0%)	70 (30·4%)	99 (41·4%)	90 (37·2%)	102 (39·4%)
	Level 3	8 (2·4%)	12 (3·6%)	60 (18·1%)	61 (18·3%)	68 (28·2%)	93 (36·0%)	78 (33·9%)	66 (27·6%)	49 (20·2%)	51 (19·7%)
**Usual Activity**	Level 4	3 (0·9%)	6 (1·8%)	96 (28·9%)	112 (33·6%)	53 (22·0%)	48 (18·6%)	21 (9·1%)	18 (7·5%)	15 (6·2%)	11 (4·2%)
	Level 5	3 (0·9%)	5 (1·5%)	141 (42·5%)	134 (40·2%)	45 (18·7%)	29 (11·2%)	13 (5·7%)	6 (2·5%)	8 (3·3%)	2 (0·8%)
	Missing	2 (0·6%)	1 (0·3%)	(0·0%)	(0·0%)	(0·0%)	(0·0%)	2 (0·9%)	(0·0%)	1 (0·4%)	(0·0%)
	p-value*	0·639		0·604		0·045		0·061		0·239	

*Fisher's exact test

Baseline analyses of participants at 16 weeks primary endpoint

Table 11 shows the comparison between the overall sample and the participants that were analysed at the 16 week primary endpoint. The results show the baseline characteristics between these samples are very similar, therefore the analysed sample at the primary endpoint reflect the general participants in the overall trial.

Table 11: Baseline Characteristics

**Baseline Characteristics**	**Sample in Primary Analysis**	**Sample not in Primary analysis**	**Overall Trial Sample**
	**(n = 502)**	**(n = 167)**	**(n = 669)**
**Gender, N (%)**			
Female	297 (59)	84 (50)	381 (57)
Male	205 (41)	83 (50)	288 (43)
**Ethnicity, N (%)**			
Asian	17 (3)	10 (6)	27 (4)
Black/African/Caribbean	20 (4)	9 (5)	29 (4)
Mixed	6 (1)	9 (5)	15 (2)
Other	6 (1)	7 (4)	13 (2)
White	450 (90)	131 (78)	581 (87)
**Pre-injury OMAS, mean (SD)**	94.6 (13)	89.8 (23)	93.4 (16)
**Baseline (post-injury) OMAS, mean (SD)**	21.1 (17)	20.4 (18)	21.0 (17)
**Age, mean (SD)**	49 (16)	40 (16)	46 (17)
**Age Group, N (%)**			
49 and Under	246 (49)	125 (75)	371 (55)
Over 49	256 (51)	42 (25)	298 (44)
**BMI, kg/m^2, mean (SD)**	28.4 (6)	28.4 (6)	28.4 (6)
**Mechanism of injury, N (%)**			
Low energy fall	323 (64)	105 (63)	428 (64)
High energy fall	83 (17)	26 (16)	109 (16)
Road traffic accident	17 (3)	9 (5)	26 (4)
Crush Injury	1 (0.2)	1 (1)	2 (0.3)
Sports Injury	42 (8)	7 (4)	49 (7)
Other	42 (8)	19 (11)	61 (9)
**Side of injury, N (%)**			
Right	243 (48)	85 (51)	328 (49)
Left	256 (51)	81 (49)	337 (50)
**Malleolus Involvement, N (%)**			
Lateral	468 (93)	156 (93)	624 (93)
Medial	138 (27)	56 (34)	194 (29)
Posterior	85 (17)	35 (21)	120 (18)
**Fracture Management, N (%)**			
Operative	271 (53)	93 (56)	364 (54)
Non-Operative	231 (46)	74 (44)	305 (46)
**Advised weight bearing status, N (%)**			
Full	160 (32)	59 (35)	219 (33)
Partial	111 (22)	29 (17)	140 (21)
None	226 (45)	77 (46)	303 (45)
**Concurrent Injuries, N (%)**			
No	457 (91)	153 (92)	624 (93)
Yes	37 (7)	8 (5)	45 (7)
**Regular smoker, N (%)**			
No	425 (85)	102 (61)	527 (79)
Yes	75 (15)	60 (36)	135 (20)
**Alcohol units (per week), N (%)**			
0-7 units	323 (64)	115 (69)	438 (65)
8-14 units	98 (20)	23 (14)	121 (18)
15-21 units	46 (9)	18 (11)	64 (10)
>21 units	34 (7)	10 (6)	44 (7)
**Taking other medication, N (%)**			

Steroids	21 (4)	3 (2)	24 (4)
Any other medications	337 (67)	102 (61)	439 (66)
**Diagnosis prior to injury, N (%)**			
Diabetes	24 (5)	8 (5)	32 (5)
Lower Limb Fracture (last 12 months)	5 (1)	0 (0)	5 (1)
Injury to lower limb (last 12 months)	6 (1)	0 (0)	6 (1)

Table 12: Baseline characteristics of participants in primary analysis by intervention group

	**Cast**	**Removable Brace**	**Overall**
	**(n = 242)**	**(n = 260)**	**(n = 502)**
**Gender, N (%)**			
Female	148 (61)	149 (57)	297 (59)
Male	94 (39)	111 (43)	205 (41)
**Ethnicity, N (%)**			
Asian	11 (5)	6 (2)	17 (3)
Black/African/Caribbean	10 (4)	10 (4)	20 (4)
Mixed	2 (1)	4 (2)	6 (1)
Other	2 (1)	4 (2)	6 (1)
White	216 (89)	234 (90)	450 (90)
**Pre-injury OMAS, mean (SD)**	95.1 (12)	94.1 (14)	94.6 (13.2)
**Baseline (post-injury) OMAS, mean (SD)**	20.5 (16.2)	21.8 (17.7)	21.1 (17.0)
**Age, mean (SD)**	49.5 (16.4)	47.9 (16.2)	48.7 (16.2)
**Age Group, N (%)**			
49 and Under	116 (48)	130 (50)	246 (49)
Over 49	126 (52)	130 (50)	256 (51)
**BMI, kg/m^2, mean (SD)**	27.9 (5.4)	28.8 (6.4)	28.4 (6)
**Mechanism of injury, N (%)**			
Low energy fall	154 (64)	169 (65)	323 (64)
High energy fall	33 (14)	50 (19)	83 (17)
Road traffic accident	10 (4)	7 (3)	17 (3)
Crush Injury	1 (0.4)	0 (0)	1 (0.2)
Sports Injury	22 (9)	20 (8)	42 (8)
Other	25 (10)	17 (7)	42 (8)
**Side of injury, N (%)**			
Right	118 (49)	125 (48)	243 (48)
Left	124 (51)	132 (51)	256 (51)
**Malleolus Involvement, N (%)**			
Lateral	225 (93)	243 (93)	468 (93)
Medial	75 (31)	63 (24)	138 (27)
Posterior	42 (17)	43 (17)	85 (17)
**Fracture Management, N (%)**			
Operative	130 (53)	141 (53)	271 (53)
Non-Operative	112 ((46)	119	231 (46)
**Advised weight bearing status, N (%)**			
Full	81 (33)	79 (30)	160 (32)
Partial	48 (20)	63 (24)	111 (22)
None	112 (46)	114 (44)	226 (45)
**Concurrent Injuries, N (%)**			
No	215 (89)	242 (93)	457 (91)
Yes	23 (2)	14 (5)	37 (7)
**Regular smoker, N (%)**			
No	207 (86)	218 (84)	425 (85)
Yes	35 (14)	40 (15)	75 (15)
**Alcohol units (per week), N (%)**			
0-7 units	167 (69)	156 (60)	323 (64)
8-14 units	37 (15)	61 (23)	98 (20)

15-21 units	24 (10)	22 (8)	46 (9)
>21 units	14 (6)	20 (8)	34 (7)
**Taking other medication, N (%)**			
Steroids	12 (5)	9 (4)	21 (4)
Any other medications	166 (69)	171 (66)	337 (67)
**Diagnosis prior to injury, N (%)**			
Diabetes	12 (5)	12 (5)	24 (5)
Lower Limb Fracture (last 12 months)	3 (1)	2(1)	5 (1)
Injury to lower limb (last 12 months)	2 (1)	4 (2)	6 (1)
